# Effects of Geostrophic Kinetic Energy on the Distribution of Mesopelagic Fish Larvae in the Southern Gulf of California in Summer/Fall Stratified Seasons

**DOI:** 10.1371/journal.pone.0164900

**Published:** 2016-10-19

**Authors:** Fernando Contreras-Catala, Laura Sánchez-Velasco, Emilio Beier, Victor M. Godínez, Eric D. Barton, Eduardo Santamaría-del-Angel

**Affiliations:** 1 Departamento de Plancton y Ecología Marina, Centro Interdisciplinario de Ciencias Marinas-Instituto Politécnico Nacional, La Paz, Baja California Sur, México; 2 Centro de Investigación Científica y de Educación Superior de Ensenada, Unidad La Paz, La Paz, Baja California Sur, México; 3 Departamento de Oceanografía Física, Centro de Investigación Científica y de Educación Superior de Ensenada, Ensenada, Baja California, México; 4 Departamento de Oceanografía, Instituto Investigaciones Marinas (CSIC), Vigo, España; 5 Facultad de Ciencias Marinas, Universidad Autónoma de Baja California, Ensenada, Baja California, México; Helmholtz-Zentrum fur Ozeanforschung Kiel, GERMANY

## Abstract

Effects of geostrophic kinetic energy flux on the three-dimensional distribution of fish larvae of mesopelagic species (*Vinciguerria lucetia*, *Diogenichthys laternatus*, *Benthosema panamense* and *Triphoturus mexicanus*) in the southern Gulf of California during summer and fall seasons of stronger stratification were analyzed. The greatest larval abundance was found at sampling stations in geostrophic kinetic energy-poor areas (<7.5 J/m^3^), where the distribution of the dominant species tended to be stratified. Larvae of *V*. *lucetia* (average abundance of 318 larvae/10m^2^) and *B*. *panamense* (174 larvae/10m^2^) were mostly located in and above the pycnocline (typically ~ 40 m depth). In contrast, larvae of *D*. *laternatus* (60 larvae/10m^2^) were mainly located in and below the pycnocline. On the other hand, in sampling stations from geostrophic kinetic energy-rich areas (> 21 J/m^3^), where mesoscale eddies were present, the larvae of the dominant species had low abundance and were spread more evenly through the water column, in spite of the water column stratification. For example, in a cyclonic eddy, *V*. *lucetia* larvae (34 larvae/10m^2^) extended their distribution to, at least, the limit of sampling 200 m depth below the pycnocline, while *D*. *laternatus* larvae (29 larvae/10m^2^) were found right up to the surface, both probably as a consequence mixing and secondary circulation in the eddy. Results showed that the level of the geostrophic kinetic energy flux affects the abundance and the three-dimensional distribution of mesopelagic fish larvae during the seasons of stronger stratification, indicating that areas with low geostrophic kinetic energy may be advantageous for feeding and development of mesopelagic fish larvae because of greater water column stability.

## Introduction

Mesoscale structures, like eddies and upwelling, typically associated with processes of mixing, convergence and divergence, are areas where the geostrophic kinetic energy is high [1–3]. Enrichment processes in these areas are enhanced by the triggering of primary and secondary productivity as nutrients are introduced into the photic zone [4,5]. Numerous studies have described qualitative relationships between mesoscale eddies and phytoplankton [6–8], and eddies with zooplankton [9–11]. Nevertheless, few studies have quantified and related the geostrophic kinetic energy and/or stratification with the distribution of planktonic organisms.

Piontkovski *et al*. [3] reported that the highest spatial heterogeneity of zooplankton biomass is found in regions of the highest available potential energy, associated with mesoscale eddy fields. Ladd *et al*. [12]. observed the highest chlorophyll *a* concentrations in areas influenced by eddies with high eddy kinetic energy in the Gulf of Alaska, suggesting that kinetic energy may be valuable for predicting phytoplankton blooms in this region. Nieto *et al*. [13] noted that high values of eddy kinetic energy were favorable for the development of sardine eggs in waters advected by eddies and filaments in the southern and central California.

The Gulf of California is a narrow, semi-enclosed and highly productive sea [14–16], which connects at the south with the Pacific Ocean. The surface circulation reverses seasonally from cyclonic in summer to anticyclonic in winter [17,18]. The Gulf is characterized by high stratification during the summer and autumn *vs* a deep surface mixing layer extending to about 100 m depth during winter and early spring [18,19]. The circulation has also a strong mesoscale component, related to the common occurrence of cyclonic and anticyclonic eddies evident in satellite infrared, color images and drifter trajectories [20–23].

Several authors [21,24,25] have described the mechanisms of eddy generation in the southern Gulf of California. They reported that the interaction of the poleward-flowing Mexican Coastal Current with specific topographic irregularities (capes at Topolobampo and Cabo Lobos), frequently generated eddies by inducing baroclinic instabilities and that this mechanism could be strengthened by the arrival of coastal trapped waves of equatorial origin. This suggests that the southern Gulf of California and the adjacent Pacific are energetic areas of mesoscale activity. Until now, no multidisciplinary studies have examined the impact of geostrophic kinetic energy flux and/or strength of stratification, as indicated by potential energy anomaly, on the planktonic organisms.

A good biological indicator for observing the role of the geostrophic kinetic energy flux and potential energy anomaly on the zooplankton organisms may be the larvae of the common mesopelagic species (*Vinciguerria lucetia*, *Diogenichthys laternatus*, *Benthosema panamense* and *Triphoturus mexicanus*). Because of their high abundances and widespread distributions, they are important components of the pelagic ecosystem in the Northeastern Pacific Ocean, including the Gulf of California [26–28]. In the Gulf, these larvae have been found to have a strongly heterogeneous distribution from the Midriff Archipelago Region to its southern entrance [29–31].

Although there are few studies of their depth dependence, it has been seen that these species tend to exhibit vertical gradients in abundance. *V*. *lucetia* and *B*. *panamense* larvae have higher abundances in the surface layer, decreasing with depth and larvae of *D*. *laternatus* and *T*. *mexicanus* have higher abundance in the layer from ~ 200 to 100 m depth, decreasing toward the surface [30,32–34]. These studies on the vertical distribution of fish larvae and of others on mesoscale physical processes, like mesoscale eddies [21,24,25], indicate that when stratification is strong (high potential energy anomaly), and geostrophic kinetic energy is low (absence of mesoscale eddies), the vertical distribution of larvae of mesopelagic species will be stratified. In contrast, where the geostrophic kinetic energy is high (presence of mesoscale eddies), the larvae will be spread through more of the water column by mixing processes and vertical advection associated with secondary circulation within the eddies.

In this context, the goal of the present paper is to explore the effects of more energetic areas on the three-dimensional distribution of fish larvae of the four abundant mesopelagic species (*V*. *lucetia*, *D*. *laternatus*, *B*. *panamense and T*. *mexicanus*) in the southern Gulf of California and its entrance during the summer/fall seasons of strongest stratification. A statistical comparison of the distribution of larvae of these four species in areas of low mesoscale activity, i.e. geostrophic kinetic energy-poor areas, and of strong mesoscale activity, i.e. geostrophic kinetic energy-rich areas in stratified conditions, was made. It seems likely that the effects of mesoscale disturbance on the mesopelagic fish larvae here might be similar to those on other zooplankton organisms in other marine regions.

## Materials and Methods

### Sampling strategy and data base

**Fig 1:**
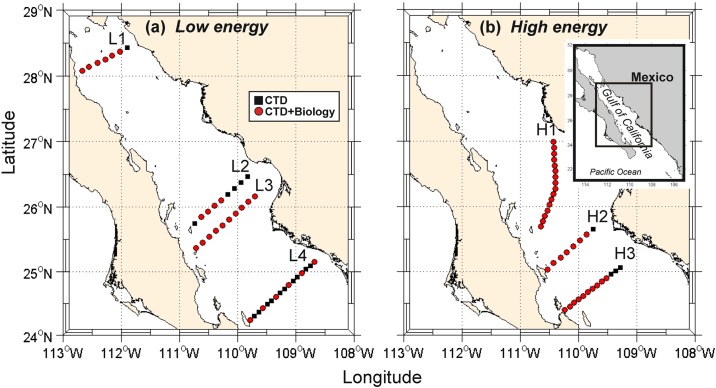
Location of the study region showing sampling stations in selected transects during five cruises. (a) Stations with low geostrophic kinetic energy flux (Transects: L1–L4) and b) stations with high geostrophic kinetic energy flux (Transects: H1–H3) in the southern Gulf of California. Black squares, CTD data only. Red circles, CTD and zooplankton data.

The bulk of the data to be discussed here is from five research cruises made during August 2005, October 2007, July 2010, July 2011 and April-May 2012, in the southern zone of the Gulf of California; periods where the stratification is high, except in April-May when the surface mixed layer was weakening with the onset of annual stratification (Table 1). The CTD and biological sampling stations of transects analyzed in each cruise are shown in different symbols in Fig 1a and 1b.

**Table 1:** General information of the oceanographic cruises made in the southern Gulf of California.

Cruise date	August 2005	July 2010	July 2010	April-May 2012	July 2011	July 2010	October 2007
Transects	L1	L2	L3	L4	H1	H2	H3
Days sampling	2	2	3	3	4	2	4
Zooplankton sampling strata (m)	0–50	0–15	0–50	0–50	0–17	0–15	0–50
		15–30			17–34	15–30	
		30–45			34–51	30–45	
	50–100	50–100	50–100	50–100	50–100	50–100	50–100
	100–150	100–150	100–150	100–150	100–150	100–150	100–150
	150–200	150–200	150–200	150–200	150–200	150–200	150–200
Zooplankton sampling stations	7	9	10	16	16	8	13
Mesoscale structure					CE	CE	AE

CE, Cyclonic eddy; AE, Anticyclonic eddy

It is important to mention that the samples were not obtained in protected natural areas or national parks; and the species that supporting this study are not endangered or protected species in according to Comisión Nacional para el Conocimiento y uso de la Biodiversidad México (CONABIO) http://www.biodiversidad.gob.mx/especies/especies_enriesgo/buscador_especies/espRiesgo.php.

The data supporting this manuscript come from a database created by a group of multidisciplinary researchers, led by the authors of this paper, mainly L. Sánchez-Velasco and E. Beier. The data are not totally available because there are other postgraduate students who are working with them on other topics for their thesis project. However we supply supplementary material with the data that supports the conclusions of this study, as PlosOne requires (S1 and S2 Tables). In addition, the basic data obtained in each cruise may be consulted in http:/usuario.cicese.mx/~mxcali/.

In the October 2007, July 2010 and July 2011cruises (Fig 1b), mesoscale eddies were detected (see details in [30,32,34]). Their position was monitored for 3 months prior to the start of each cruise, using daily satellite images of chlorophyll *a* concentrations from the Moderate Resolution Imaging Spectroradiometer (MODIS) sensors on board the Aqua satellite (Satellite images come from NASA Earth Observatory “public domain”: http://earthobservatory.nasa.gov/). The images, with a spatial resolution of 1 km/pixel (Local Area Cover (LAC)), were processed using Level 1B data by NASA (http://oceancolor.gsfc.nasa.gov with July 2015 flag quality data) to Level 2 with SeaDAS, version 5.5, using the OC3M v4 algorithm [35]. Level 3 imagery was constructed in the Universidad Autónoma de Baja California with an equidistant cylindrical projection.

The hydrographic structure of transects made during these cruises has been documented previously [30,32,34]. With the aid of the satellite images, transects were divided into two sets (Fig 1a and 1b). The first included all those that crossed mesoscale eddies (H1, H2 and H3), while the second consisted of those with weak mesoscale influence (L1, L2, L3 and L4). Transect details (e.g., number of stations, periods and year) are shown in Table 1.

At all sampling stations (Fig 1), temperature and conductivity profiles down to 1000 m (or near the bottom if shallower) were measured with a factory-calibrated CTD (SeaBird SBE-911 plus), with primary and secondary sensors and a sampling rate of 24 Hz. Dissolved oxygen (DO, mL/L) and fluorescence (mg/m^3^) sensors (SBE43 and SeaPoint, respectively) were also included. The data were processed and averaged to 1 db as documented by Godínez *et al*. [36,37]. Conservative Temperature (Θ, °C), Absolute Salinity (*S*_*A*_, g/ kg) and Density Anomaly (σ_Θ_, kg/m^3^) were calculated from *in situ* temperature and practical salinity with the TEOS-10 (Thermodynamic Equation of Seawater-2010) software, which was downloaded from http://www.TEOS-10.org [38,39].

The geostrophic velocity relative to the minimum common sampling depth of pairs of stations was calculated from objectively mapped Θ (°C) and S_*A*_ distributions. A standard objective mapping interpolation was used, using a classic Gaussian correlation function with relative errors of 0.1, horizontal length scale of 70 km and vertical scale of 30 km. The horizontal scale is about twice the internal radius of deformation for the region [40], which ensures that geostrophic flow is resolved by the smoothed hydrographic data. The ocean surface mixed layer depth was calculated following the methodology of Kara *et al*. [41] which consists in a gradient-based criterion having a fixed temperature difference of 0.8°C.

Geostrophic Kinetic Energy flux [42](J/m^3^) can be understood as the mean geostrophic kinetic energy flux between stations along each transect and it was calculated as:

$${KE}_{g}=\frac{1}{h}\int_{-h}^{0}\rho v_{g}^{2}dz$$ (1)

where *v*_*g*_ is the geostrophic velocity normal to each section, *h* is the depth as defined above, *ρ* is the water density and *dz* is the distance between vertical samples. *KE*_*g*_ can be understand as the mean geostrophic kinetic energy flux at each cast along each transect and to 300m depth. Geostrophic Kinetic Energy flux includes the contribution of eddies, filaments and others mesoscale processes [43], but we consider that when an eddy is present, the major part of the Geostrophic Kinetic Energy flux results from the eddy dynamics.

Potential energy anomaly (*φ*), defined as the amount of work per unit volume required to redistribute the mass in a complete mixing of a water column to a specified depth [44], was calculated in J/m^3^.

$$\varphi =\frac{1}{h}\int_{-h}^{0}(\bar{\rho }-\rho )gz^{}\;dz;\;\bar{\rho }=\frac{1}{h}\int_{-h}^{0}\rho dz;$$ (2)

where *z* is the vertical coordinate (positive upwards), *ρ* (*z*) is the potential density calculated from in situ densities in a water column of depth *h*. The anomaly of potential energy is positive for a stably stratified water column and it is negative for an unstably stratified water column. Physically, *φ* gives the amount of energy per volume necessary to bring about complete vertical mixing over a specific depth interval. The specified depth h for this study is 300 m, to cover the pycnocline at all locations and times of year within the study area. The potential energy anomaly of Simpson *et al*. [44] can be understood as a measure of water column stratification.

Transect-averaged values for these parameters range from 5–30 J/m^3^ for geostrophic kinetic energy and 400–1000 J/m^3^ for potential energy anomaly (Table 2). “Low” and “high” therefore are very different for the two variables, which clearly are not directly comparable even though they are expressed in the same units.

**Table 2:** Values of geostrophic kinetic energy flux and potential energy anomalies (J/m^3^) data referred to 300 m depth (see position of transects in Figs 2–8).

Date of cruise	Transects	Mesoscale structure	Geostrophic kinetic energy flux (J/m3)	Potential energy anomaly (J/m^3^)
August 2005	L1		4.8	1074.0
July 2010	L2		5.2	775.8
July 2010	L3		7.2	799.4
April 2012	L4		6.9	422.65
July 2011	H1	Cyclonic eddy	33.2	928.03
July 2010	H2	Cyclonic eddy	21.3	744.19
October 2007	H3	Anticyclonic eddy	13.6	1097.68

Stratified zooplankton hauls were made during day and night in different depth strata selected according to the hydrographic structure and the logistics available in each cruise (see Table 1). During July 2010 (L2, H1 and H2) and July 2011 (H1), the hauls were made in every 17 m layer down to the thermocline, and in every 50 m layer from the thermocline down to 200 m depth. In August 2005 (L1), October 2007 (H3) and April-May 2012 (L4), the hauls were every 50 m layer from the surface to 200 m depth. Opening–closing conical zooplankton nets, with a 50 cm mouth diameter, 250-cm net length and 505μm mesh size were used (http://www.generaloceanic.com). The closed net was situated at the bottom of the stratum to be sampled, then it was opened with a manual brass messenger, and the haul was initiated. When the top of the sampling layer was reached, the net was closed with another messenger and the haul ended. This system avoids sample contamination with organisms from other strata. To ensure accurate sampling of each depth stratum, the depth of the net was calculated by the cosine of the wire angle method following the standard specifications of Smith and Richardson [45]. This stratified sampling technique has been used successfully in previous studies (e.g., [34,46]).

The volume of filtered water was calculated using calibrated flowmeters placed in the mouth of each net. Samples were fixed with 5% formalin buffered with sodium borate. Zooplankton biomass, estimated by the displacement volume [47], was standardized to mL/1000 m^3^. Fish larvae were removed from all samples and the *V*. *lucetia*, *D*. *laternatus*, *B*. *panamense* and *T*. *mexicanus* were identified as in Moser [48]. The developmental stage was determined in relation to notochord flexion following the criteria of Kendall *et al*. [49] and only larval fish pre-flexion were selected. Fish larval abundance was standardized to number of larvae per 10 m^2^ [45,50].

### Data analysis

Two biological matrices (not shown), one for each set of transects (Table 1), were constructed. To assess the statistical significance of differences of the total larval abundance between daytime and nighttime, the non-parametric Mann–Whitney test was used [51,52]. A cluster analysis based on a species abundance matrix *vs* samples [53] was applied to determine three-dimensional larval fish habitats (or groups of samples), and their characteristic species (Table A in S1 and S2 Tables). An agglomerative dendrogram was constructed on the basis of a triangular similarity matrix (Bray-Curtis dissimilarity measure; see [52]) using Flexible beta (*β* = -0.25) linkage on fourth-root transformed larval abundance to minimize the effect of outlier values [54]. The scaling of the dendrogram is Wishart's objective function [55], which measures the information lost at each step in hierarchical cluster analysis. As groups are fused, the amount of information decreases until all groups are fused and no information remains. The objective function can be rescaled from 0% to 100% of information [56]. To detect significant differences among the dissimilarity of the distinct larval fish habitats, a one-way ANOSIM (analysis of similarities) was applied as a test of the significance of the habitats that had been defined *a priori* by the Bray-Curtis dissimilarity measure The procedures use the difference between average ranked values of Bray-Curtis measures of dissimilarity in abundances and types of organisms among replicates between samples (*r*_*b*_) and within samples (*r*_*w*_) to give a test statistic,

$$R=\frac{r_{b}-r_{w}}{\frac{1}{4}[n\left(n-1\right)]}$$ (3)

where n is the total number of replicates summed for the 2 samples. R is scaled to lie between -1 and +1, a value of zero representing the null hypothesis of no differences among samples of the habitats [53,57].

The Olmstead–Tukey test determined hierarchies of the species in each larval fish habitat (dominant, frequent, constant and rare species) (e.g., [11,58]). This test considered the average relative abundance against the frequency of occurrence of each species [52]. Average similarity and the percentage of contribution of specific species to the identity of each habitat were determined using the Similarity Percentage (SIMPER) routine. This analysis calculates the contribution of each species (or other variable) to the observed similarity between samples. It allows identification of the species that are most important in the observed pattern of similarity. The method uses the Bray-Curtis measure of similarity, comparing in turn, each sample in Group i with each sample in Group ii. The Bray-Curtis method operates at the species level, and therefore the mean similarity between Groups *i* and *ii* can be obtained for each species (PRIMER v7; [59]).

A canonical correspondence analysis [60] was run to define the relation between environmental variables and larval fish distribution (S1 and S2 Tables), after fourth-root transformation of the standardized biological data and the matrix of environmental indicators. This matrix contained the zooplankton displacement volume (mL/1000 m^3^) of each stratum and the stratum-average values of Conservative Temperature (Θ °C), Absolute Salinity (*S*_*A*_, g/kg), chlorophyll *a* (mg/m^3^) and dissolved oxygen (mL/L).

## Results

### Hydrographic structure and circulation

#### Transects with little evidence of mesoscale eddies

Sections of all properties are plotted to the depth of 220 m, just below the maximum fish larvae sampling depth.

**Fig 2:**
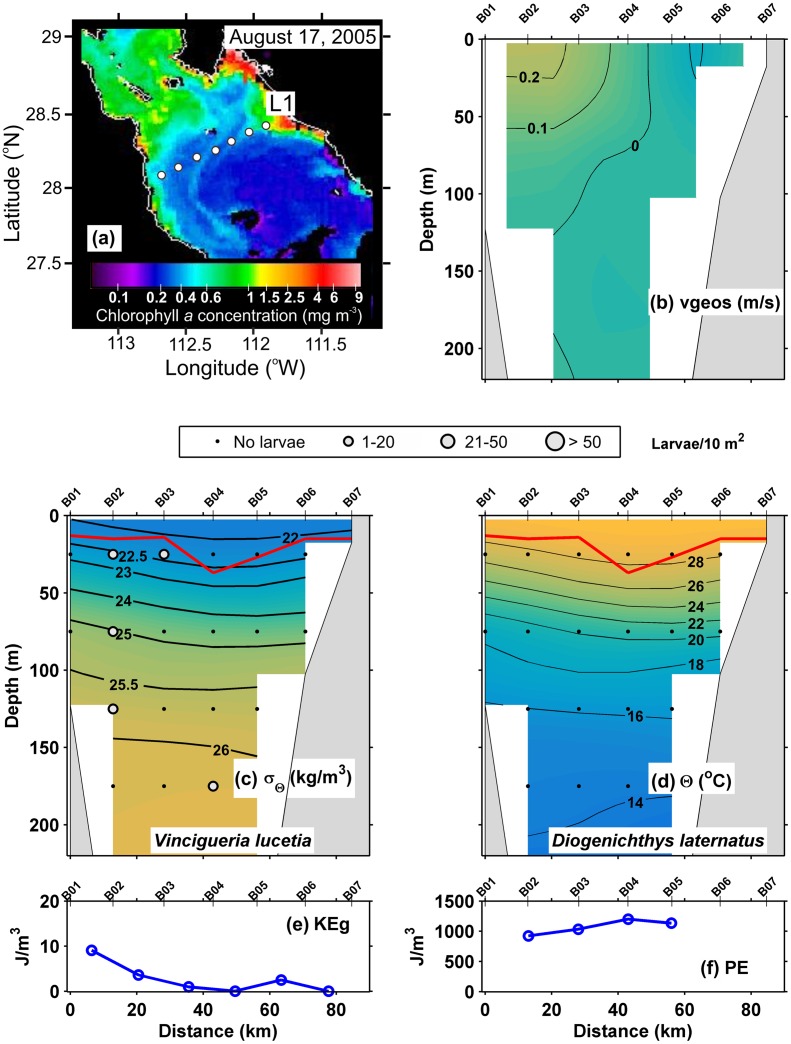
Three-dimensional distribution of fish larvae in low geostrophic kinetic energy flux transect (L1) (August, 2005). (a) Sea surface Chlorophyll *a* concentrations (MODIS-AQUA LAC) on August 17, 2005. Dots represent the stations sampled. (b) Vertical section of geostrophic velocity (m/s). (c) Vertical distribution of *Vinciguerria lucetia* larvae (larvae/10m^2^) on the density anomaly (kg/m^3^). (d) *Diogenichthys laternatus* larvae (larvae/10m^2^) on the thermal structure (Θ°C). (e) Graphs of the geostrophic kinetic energy flux (J/m^3^) and (f) potential energy anomaly (J/m^3^). The heavy red curve in Fig 2c and d, marks the surface mixed layer depth. Figures are drawn with west at the left hand side.

In the August 2005 section L1 (Fig 2a), the geostrophic velocities were < 0.1 m/s, except in an apparent coastal current near the Baja California peninsula (Fig 2b). The pycnocline was defined as the layer of strongest vertical density gradient between the 25 and 23 kg/m^3^ isopycnals at ~ 50 m depth, beneath the surface mixed layer, which fluctuated between ~ 10 and 25 m depth (Fig 2c). The thermal structure (Fig 2d) revealed a shallow thermocline between the 25 and 20°C isotherms, coinciding with the pycnocline. The geostrophic kinetic energy flux (View Eq (2)) had an average value of 4.8 J/m^3^ (Table 2), showing low values along the transect (Fig 2e), except near to the peninsula coast (~ 10 J/m^3^). The average of the potential energy anomaly was of 1074 J/m^3^ (Table 2) being > 900 J/m^3^ along of the transect (View Eq (3)) (Fig 2f).

**Fig 3:**
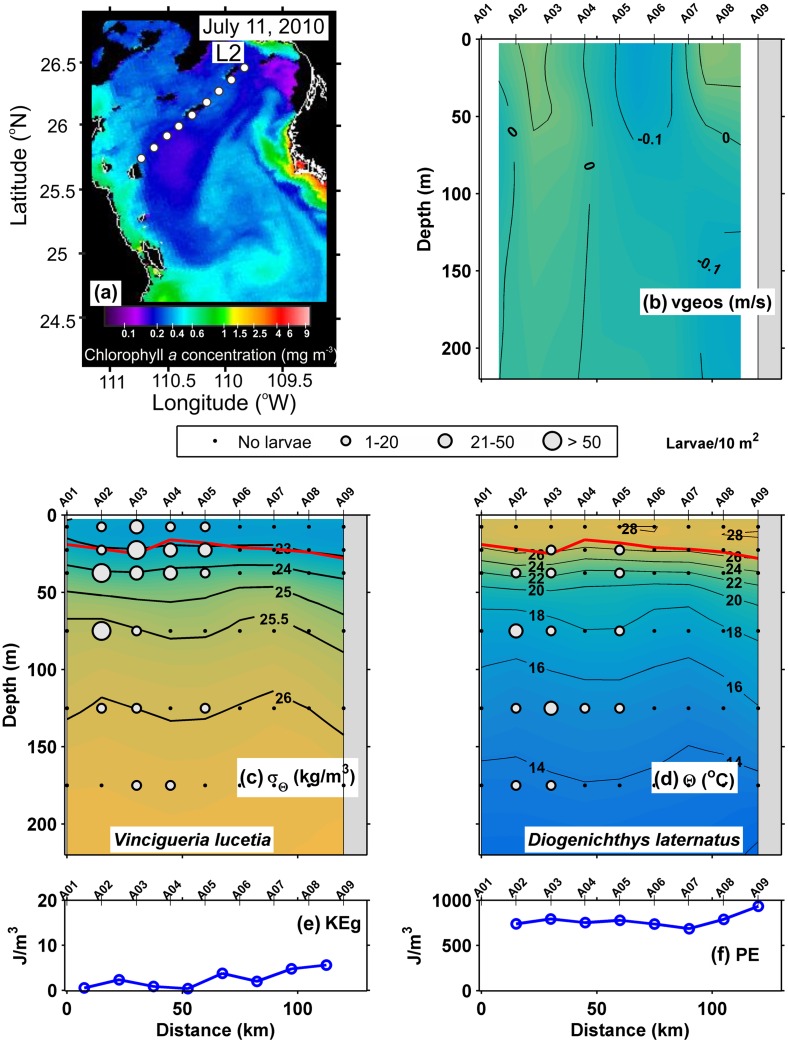
Three-dimensional distribution of fish larvae in low geostrophic kinetic energy flux transect (L2) (July, 2010). (a) Sea surface Chlorophyll *a* concentrations (MODIS-AQUA LAC) on July 11, 2010. Dots represent the stations sampled. (b) Vertical section of geostrophic velocity (m/s). (c) Vertical distribution of *Vinciguerria lucetia* larvae (larvae/10m^2^) on the density anomaly (kg/m^3^). (d) *Diogenichthys laternatus* larvae (larvae/10m^2^) on the thermal structure (Θ°C). (e) Graphs of the geostrophic kinetic energy flux (J/m^3^) and (f) potential energy anomaly (J/m^3^). The heavy red curve in Fig 3c and d, marks the surface mixed layer depth. Figures are drawn with west at the left hand side.

**Fig 4:**
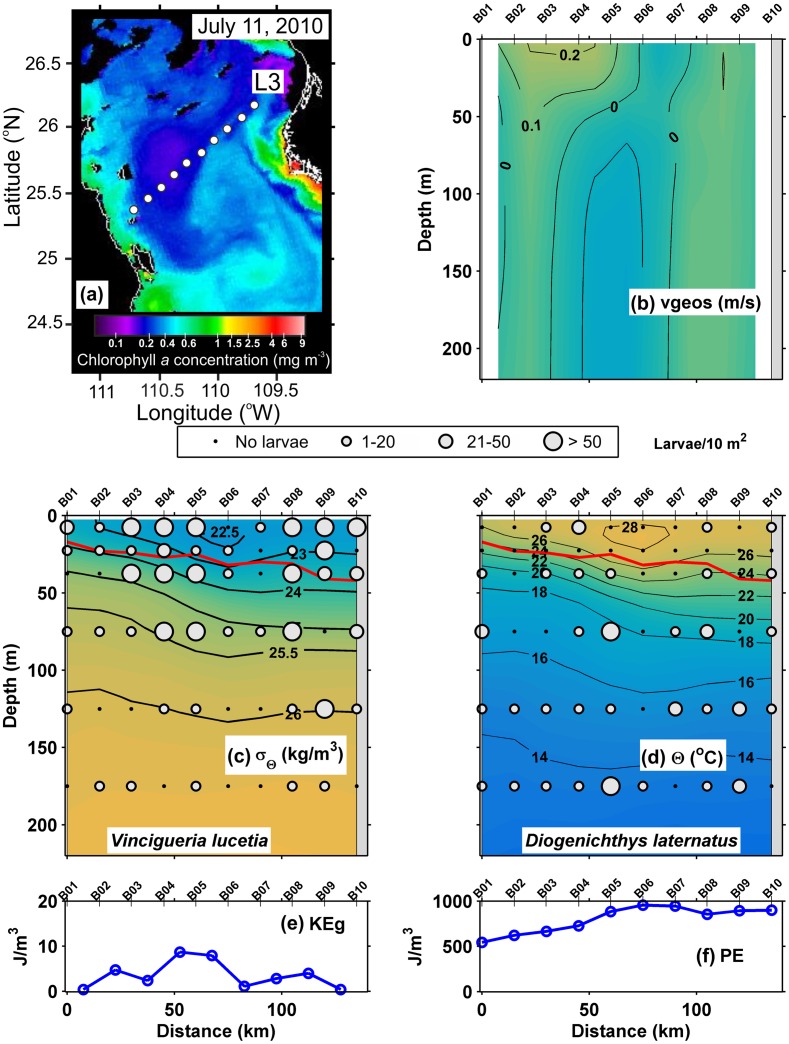
Three-dimensional distribution of fish larvae in low geostrophic kinetic energy flux transect (L3) (July, 2010). (a) Sea surface Chlorophyll *a* concentrations (MODIS-AQUA LAC) on July 11, 2010. Dots represent the stations sampled. (b) Vertical section of geostrophic velocity (m/s). (c) Vertical distribution of *Vinciguerria lucetia* larvae (larvae/10m^2^) on the density anomaly (kg/m^3^). (d) *Diogenichthys laternatus* larvae (larvae/10m^2^) on the thermal structure (Θ°C). (e) Graphs of the geostrophic kinetic energy flux (J/m^3^) and (f) potential energy anomaly (J/m^3^). The heavy red curve in Fig 4c and d, marks the surface mixed layer depth. Figures are drawn with west at the left hand side.

Both transects made during July 2010, L2 and L3 respectively, indicate the pycnocline lay between the 25 and 23 kg/m^3^ isopycnals at ~ 40 m depth with surface mixed layer between ~ 15 and 20 m deep (Figs 3c and 4c), but in L3, the pycnocline and the surface mixing layer deepened to ~ 50 m depth near to the mainland coast. The thermocline, located between the 26 and 18°C isotherms, coincided with the pycnocline (Figs 3d and 4d). The geostrophic velocities were ≤ 0.1 m/s everywhere except in the line L3 near to the peninsular coast, where the speeds reached up to 0.2 m/s, possibly a jet associated with upwelling at the near-shore stations (Fig 4b). The values of geostrophic kinetic energy flux were low along both lines, with an average of 5.2 and 7.2 J/m^3^ respectively **(**Table 2), with a slight increase where the geostrophic velocities were ≤ 0.1 m/s (Figs 3e and 4e). In contrast, the potential energy anomaly values were high along of both transects, mostly > 700 J/m^3^ (Figs 3f and 4f).

**Fig 5:**
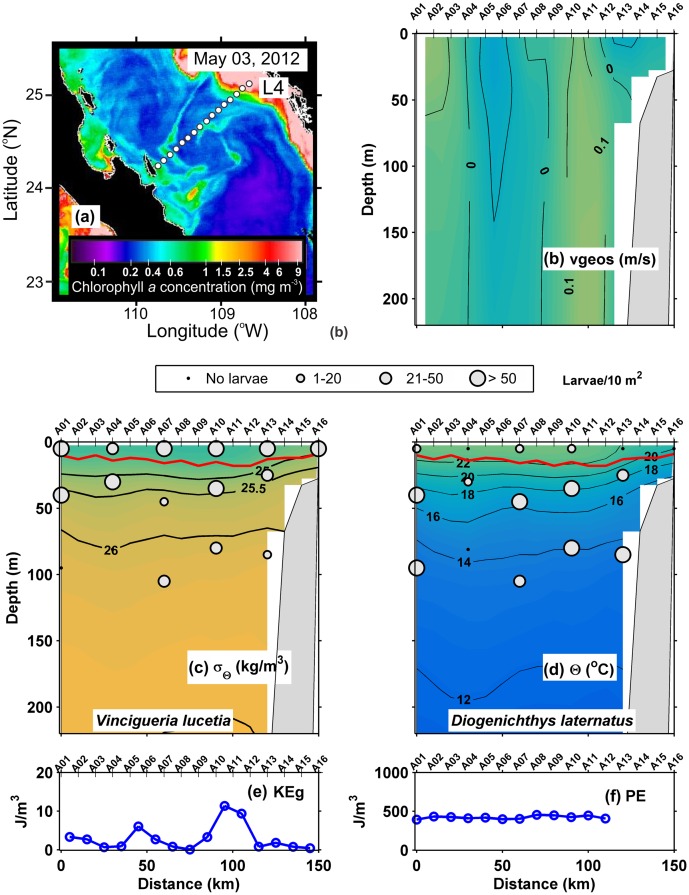
Three-dimensional distribution of fish larvae in low geostrophic kinetic energy flux transect (L4) (May, 2012). (a) Sea surface Chlorophyll *a* concentrations (MODIS-AQUA LAC) on May 03, 2012. Dots represent the stations sampled. (b) Vertical section of geostrophic velocity (m/s). (c) Vertical distribution of *Vinciguerria lucetia* larvae (larvae/10m^2^) on the density anomaly (kg/m^3^). (d) *Diogenichthys laternatus* larvae (larvae/10m^2^) on the thermal structure (Θ°C). (e) Graphs of the geostrophic kinetic energy flux (J/m^3^) and (f) potential energy anomaly (J/m^3^). The heavy red curve in Fig 5c and d, marks the surface mixed layer depth. Figures are drawn with west at the left hand side.

In the transect sampled during April-May 2012 L4 (Fig 5a), the geostrophic velocities were again weak ≤ 0.1 m/s **(**Fig 5b), the pycnocline was situated between the 25.5 and 25 kg/m^3^ isopycnals at ~ 40 m depth, and a surface mixed layer was ~ 10 m thick (Fig 5c). The thermocline lay between the 22 and 18°C isotherms, following the depth of the pycnocline (Fig 5d). The geostrophic kinetic energy flux had an average of 6.9 (J/m^3^) (Table 2), showing low values along the transect, except in locations of strongest geostrophic velocity **(**Fig 5e), while the potential energy anomaly was relativity low and constant throughout the transect with values ~ 450 J/m^3^ (Fig 5f).

#### Transects with evidence of mesoscale eddies

**Fig 6:**
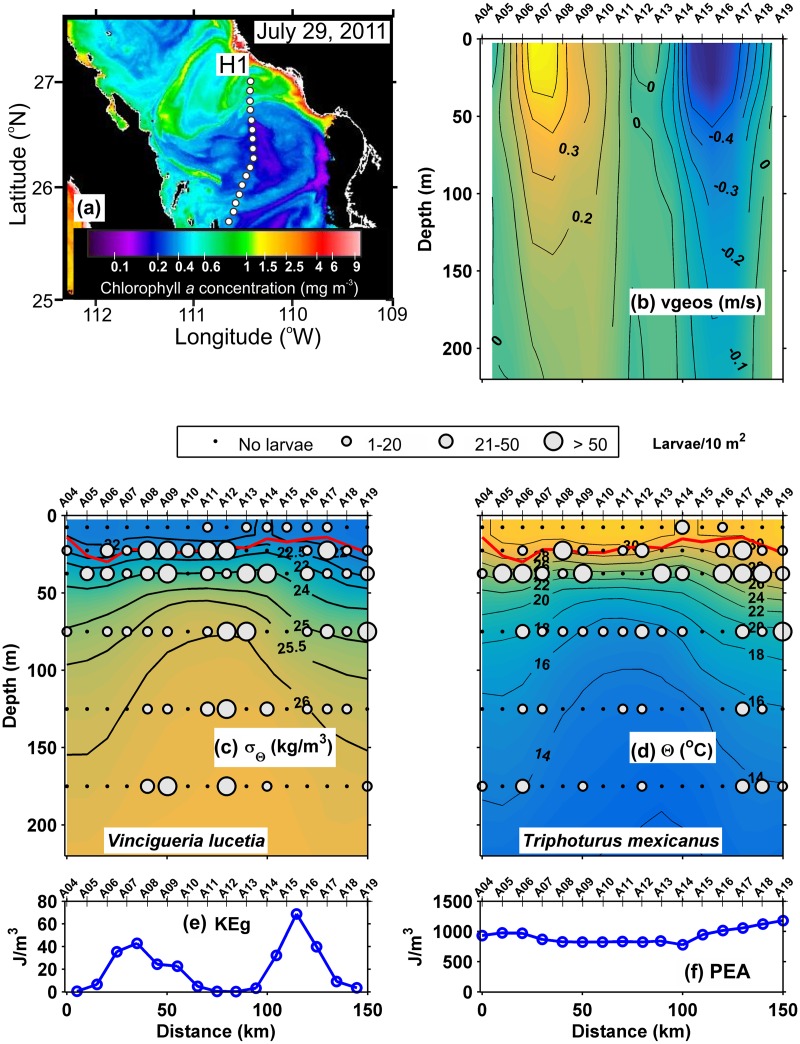
Three-dimensional distribution of fish larvae in high geostrophic kinetic energy flux transect (H1) (July, 2011). (a) Sea surface Chlorophyll *a* concentrations (MODIS-AQUA LAC) on July 29, 2011. Dots represent the sampling stations in a high energy Line (H1). (b) Vertical section of geostrophic velocity (m/s). (c) Vertical distribution of *Vinciguerria lucetia* larvae (larvae/10m^2^) on the density anomaly (kg/m^3^). (d) *Triphoturus mexicanus* larvae (larvae/10m^2^) on the thermal structure (Θ°C). (e) Graphs of the geostrophic kinetic energy flux (J/m^3^) and (f) potential energy anomaly (J/m^3^). The heavy red curve in Fig 6c and d, marks the surface mixed layer depth. Figures are drawn with south at the left hand side.

**Fig 7:**
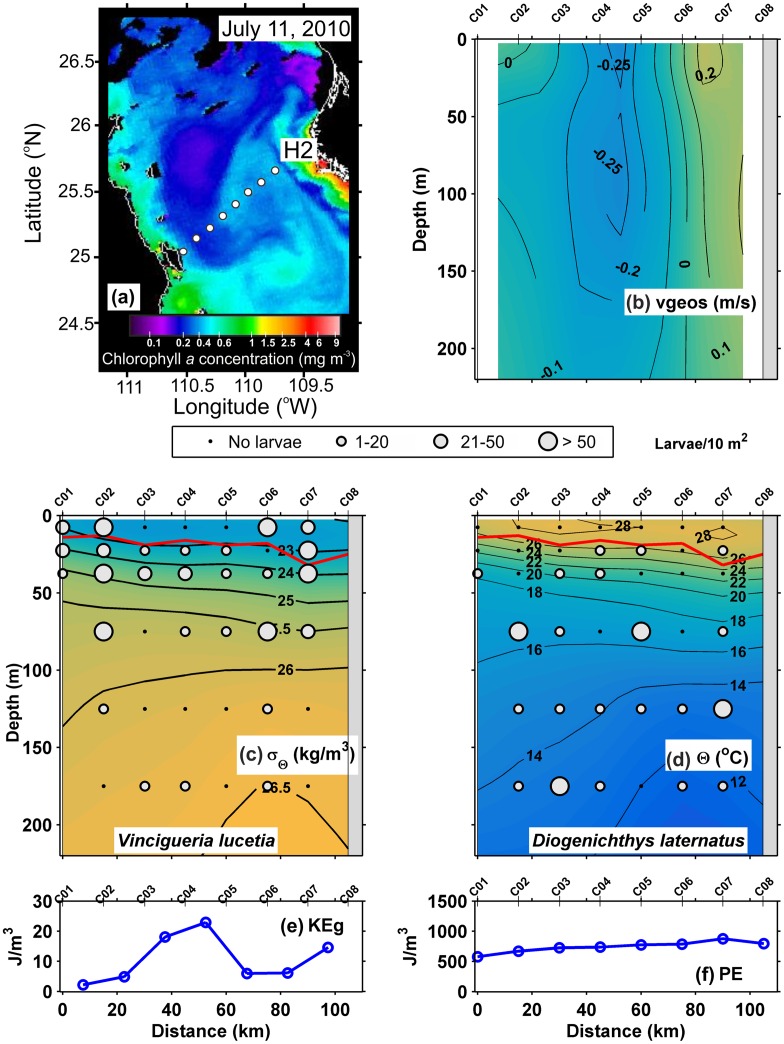
Three-dimensional distribution of fish larvae in high geostrophic kinetic energy flux transect (H2) (July, 2010). (a) Sea surface Chlorophyll *a* concentrations (MODIS-AQUA LAC) on July 11, 2010. Dots represent the sampling stations. (b) Vertical section of geostrophic velocity (m/s). (c) Vertical distribution of *Vinciguerria lucetia* larvae (larvae/10m^2^) on the density anomaly (kg/m^3^). (d) *Diogenichthys laternatus* larvae (larvae/10m^2^) on the thermal structure (Θ°C). (e) Graphs of the geostrophic kinetic energy flux (J/m^3^) and (f) potential energy anomaly (J/m^3^). The heavy red curve in Fig 7c and d, marks the surface mixed layer depth. Figures are drawn with west at the left hand side.

**Fig 8:**
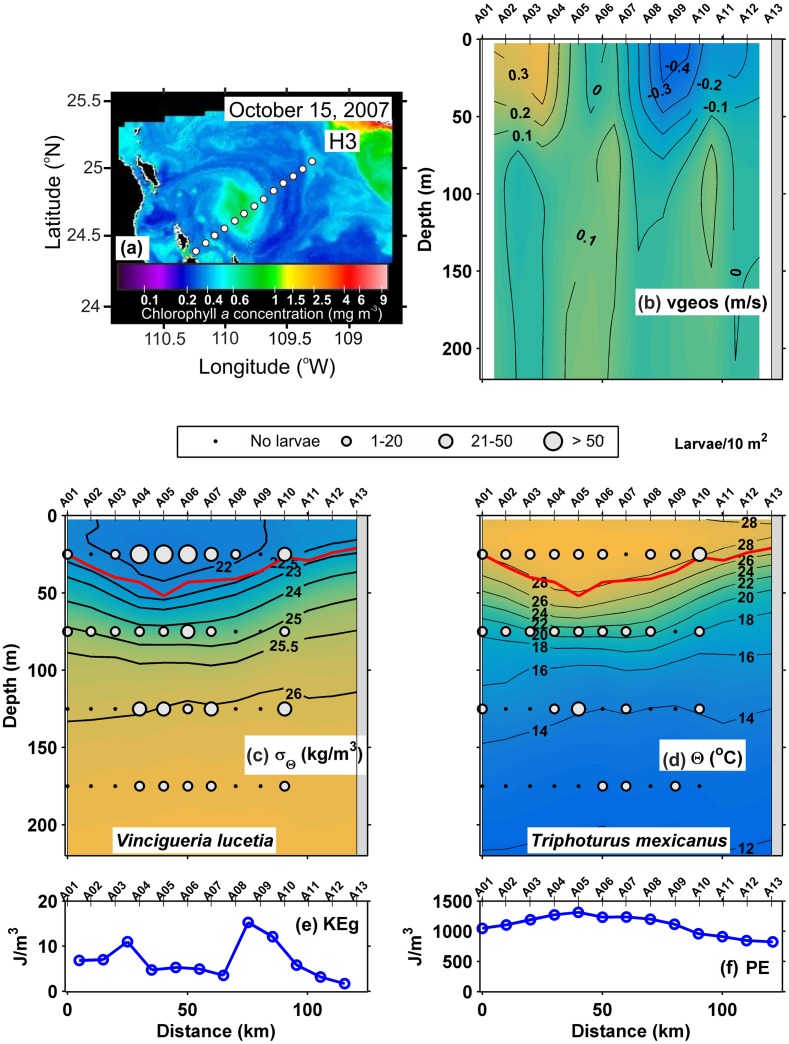
Three-dimensional distribution of fish larvae in high geostrophic kinetic energy flux transect (H3) (October, 2007). (a) Sea surface Chlorophyll *a* concentrations (MODIS-AQUA LAC) on October 15, 2007. Dots represent the sampling stations. (b) Vertical section of geostrophic velocity (m/s). Vertical distribution of (c) *Vinciguerria lucetia* larvae (larvae/10m^2^) on the density anomaly (kg/m^3^). (d) *Triphoturus mexicanus* larvae (larvae/10m^2^) on the thermal structure (Θ°C). (e) Graphs of the geostrophic kinetic energy flux (J/m^3^) and (f) potential energy anomaly (J/m^3^). The heavy red curve in Fig 8c and d, marks the surface mixed layer depth. Figures are drawn with west at the left hand side.

In transects with evidence of mesoscale eddies the sections of geostrophic velocity and hydrographic structure across mesoscale eddies observed in the Gulf are shown in Figs 6–8.

A clearly defined eddy was detected by satellite images during the July 2011 section H1 (Fig 6a). The geostrophic velocities revealed a cyclonic eddy of diameter ~ 150 km extending to > 300 m depth, with azimuthal velocities > 0.35 m/s (Fig 6b). The pycnocline, observed at ~ 50 m depth between ~25.5 and 22 kg/m^3^ isopycnals, was compressed between stations A08 and A14, where the surface mixed layer was ~ 20 m deep (Fig 6c). The isopycnals below the pycnocline presented a dome in the central part (stations A08 and A14) of the section, at all depths down to 300 m. The thermocline, defined between the 16 and 28°C isotherms showed a similar distribution to the pycnocline (Fig 6d). Details of the eddy are presented in Sanchez-Velasco *et al*. (2013). The average of geostrophic kinetic energy flux was the highest observed during our cruises (33.2 J/m^3^) (Table 2). The greatest values occurred where the geostrophic velocity was ≥ 0.3 m/s (Fig 6e). The potential energy anomaly with an average of 928 J/m^3^ was high all along the transect (Fig 6f).

In the Line H2 of July 2010, part of an eddy was sampled, as evident in Fig 7a. The geostrophic velocities (Fig 7b) showed rotation with azimuthal velocities > 0.1 m/ s, indicating the presence of a weak cyclonic eddy. The diameter of the eddy was ~ 60 km (between C04 and C07) and its depth was > 300 m. The pycnocline, observed between the 25 and 23 kg/m^3^ isopycnals, was depressed and the surface mixed layer was thickened, from ~ 25 m (C01 and C02) to ~ 50 m depth (C06 and C08). However, the 26.5 kg/m^3^ isopycnal below the pycnocline was strongly domed between stations C05 and C07, showing an elevation from 290 m at C01 to 190 m at C06 (Fig 7c). The thermal structure in Fig 7d, was similar to the isopycnals distribution and the 12°C isotherm rose from 260 m to 180 m. A more detailed hydrographic description of this eddy is provided in Contreras-Catala *et al*. (2015). The geostrophic kinetic energy flux presented a high average value (21.3 J/m^3^) (Table 2), with maximum values where the geostrophic velocity was highest (Fig 7e). The potential energy anomaly was high with an average of 744.19 J/m^3^ (Table 2), showing a slight increase to mainland coast (Fig 7f).

A small eddy was detected in the satellite image (Fig 8a) near the date of the October 2007 section H3. The geostrophic velocities (Fig 8b) showed rotation reaching azimuthal velocities > 0.25 m/s, indicating an anticyclone of ~ 90 km diameter and 70 m depth in its center. The pycnocline extended from the 25 to 23 kg/m^3^ isopycnals (Fig 8c), and the thermocline was found between the 26 and 18°C isotherms (Fig 8d). Both pycnocline and thermocline showed a central depression (between A02 and A11), where the surface mixed layer increased from ~ 20 m in the edge to 50 m depth in the center. A detailed description of this eddy can be seen in Contreras-Catala *et al*. (2012). The geostrophic kinetic energy flux had an average value of 13.6 J/m^3^ (Table 2), showing the lowest values in the eddy center (Fig 8e). In contrast, the potential energy anomaly showed the highest values in the center of the eddy ~ 1100 J/m^3^, decreasing in the margins, mainly on the side of the continental coast (Fig 8e).

### Three-dimensional distribution of mesopelagic fish larvae

#### Transects with little evidence of mesoscale eddies

**Fig 9:**
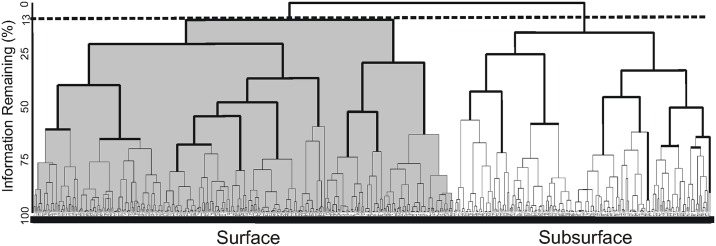
Dendrogram of larval fish samples defined by Bray-Curtis dissimilarity derived from low geostrophic kinetic energy flux transects. Fish larvae data collected in the southern of the Gulf of California.

In this section the statistical results applied to a matrix of fish larvae abundance from the transects with low mesoscale activity are presented. There were no statistically significant differences in the larval abundance between day and night hours (with 95% confidence level). The Bray-Curtis dissimilarity measure defined two larval fish habitats to a cut of 13% of the information remaining of the data set (Fig 9). These habitats were significantly different (ANOSIM: *R* = 0.4, with 95% confidence level) and were named according to their location in the water column as “Surface larval fish habitat” (grey shaded in Fig 9) and “Subsurface larval fish habitat” (unshaded in Fig 9).

The “Surface larval fish habitat”, located in and above the thermocline, was formed by 325 samples and had the highest larval average abundance with 91 larvae/10 m^2^. The dominant species were *V*. *lucetia* and *B*. *panamense*, species with a known affinity to the surface layer, with a contribution of 56 and 29% respectively in the habitat conformation (Table 3). The distribution of *V*. *lucetia* was observed in the Figs 2c, 3c, 4c and 5c. Its highest abundance was from the thermocline to the surface associated mostly with stations of low geostrophic kinetic energy, except in August 2005 (Fig 2c), when few larvae were observed. *B*. *panamense* larvae showed a similar distribution to *V*. *lucetia* larvae in most of the transects, but in August 2005 presented high abundance throughout the sampled water column (not shown).

**Table 3:** Olmstead–Tukey test, One-way ANOSIM (View Eq (3)) and SIMPER analyses between larval fish habitats (LFH) classified according to the Bray-Curtis measured in lowest geostrophic kinetic energetic zone.

Habitat	Surface				Subsurface			
Number of samples	235				207			
Mean zooplankton biomass (mL/1000m^3^)	358.5				241.2			
Mean larval abundance (larvae/10m^2^)	91				57			
Taxa	H	X	%F	%S	H	X	%F	%S
*Vinciguerria lucetia*	D	318	86	56	C	13	59	27
*Diogenichthys laternatus*	R	60	50	4	D	24	48	25
*Benthosema panamense*	D	174	76	29	D	18	55	32
*Triphoturus mexicanus*	R	45	65	11	R	14	48	16

R = 0.40, value in ANOSIM (Analysis of similarities); SIMPER, Similarity percentages.

H, hierarchy; X, mean abundance; %F, percentage of occurrence; %S, Similarity percentage (%Contribution); D, dominant; C, constant; F, frequent; R, rare.

The “Subsurface larval fish habitat” was located mainly in the thermocline and below it. This habitat was defined by 207 samples, with a larval average abundance of 57 larvae/10 m^2^. *D*. *laternatus* and *B*. *panamense* were the dominant species, which had a contribution ≥ 25% in the habitat conformation (Table 3). The *D*. *laternatus* larvae were located throughout the sampled water column, but with highest abundance below the thermocline and particularly with low geostrophic kinetic energy flux. In August 2005, they were completely absent (Figs 2d, 3d, 4d and 5d).

**Fig 10:**
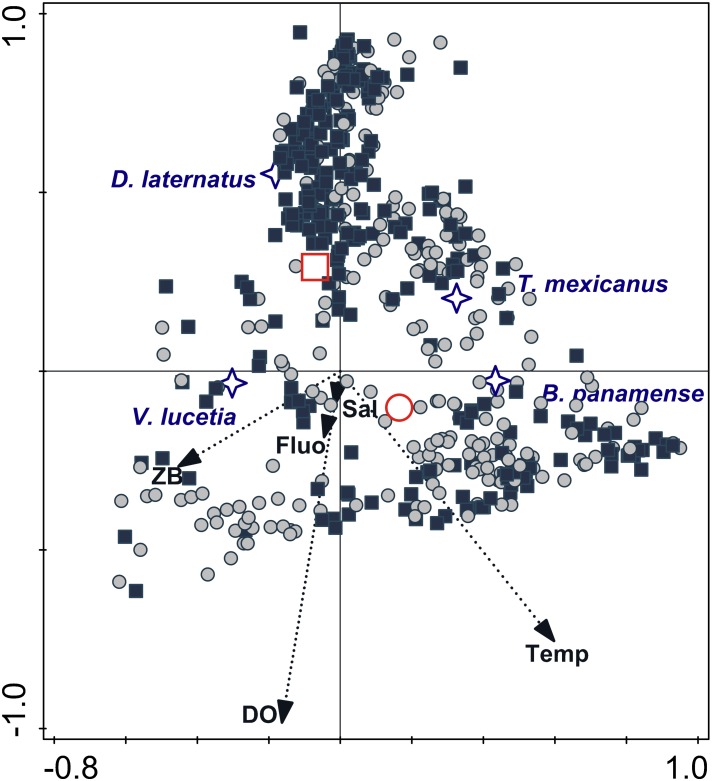
Triplot based on a Canonical Correspondence Analysis (CCA) ordination diagram from low geostrophic kinetic energy flux transects. Biological samples (squares and circles), larval fish habitat centroids (red symbols), species centroids (stars) and environmental data (arrows); first axis is horizontal and second axis vertical. Data collected on five cruises in southern Gulf of California and adjacent Pacific. Sal: Absolute salinity; ZB: zooplanktonic displacement volume; Fluo: fluorescence; Temp: Conservative temperature; DO: dissolved oxygen.

The definitions of these two larval fish habitats were apparently detected by the CCA (Fig 10), with a high correlation between variables (Pearson correlation 0.72) (Table 4). The eigenvalues of axis 1 (horizontal) and axis 2 (vertical) were 0.37 and 0.11, respectively; the eigenvalue of the axis 3 (not displayed) was 0.009. The “Surface larval fish habitat”, although present in all quadrants of the triplot, had higher frequency in the lower quadrants. This habitat was correlated with high levels of dissolved oxygen, Conservative Temperature, Absolute Salinity and zooplankton displacement volume, associating with high larval abundance of *V*. *lucetia* and *B*. *panamense*; this last species was especially correlated with the highest values of temperature. While the “Subsurface larval fish habitat” showed an inverse correlation with the environmental variables mentioned above, it was associated with high larval abundance of *D*. *laternatus*.

**Table 4:** Contribution percentage of the explanatory variables by the canonical correspondence analysis.

**Variable in the zone with lowest geostrophic kinetic energetic zone**	**Explained variance %**	**Relative contribution (%)**
Conservative temperature	14.6	39.7
Dissolved oxygen	17.7	48.3
Absolute Salinity	2.8	7.5
Zooplankton displacement biomass	0.8	2.3
Fluorescence	0.8	2.2
**Variable in the zone with highest geostrophic kinetic energetic zone**	**Explained variance %**	**Relative contribution (%)**
Conservative temperature	19	51.7
Dissolved oxygen	16.9	46
Absolute Salinity	10.3	28
Fluorescence	2.8	7.7
Zooplankton displacement biomass	2.2	6.1

#### Transects with evidence of mesoscale eddies

**Fig 11:**
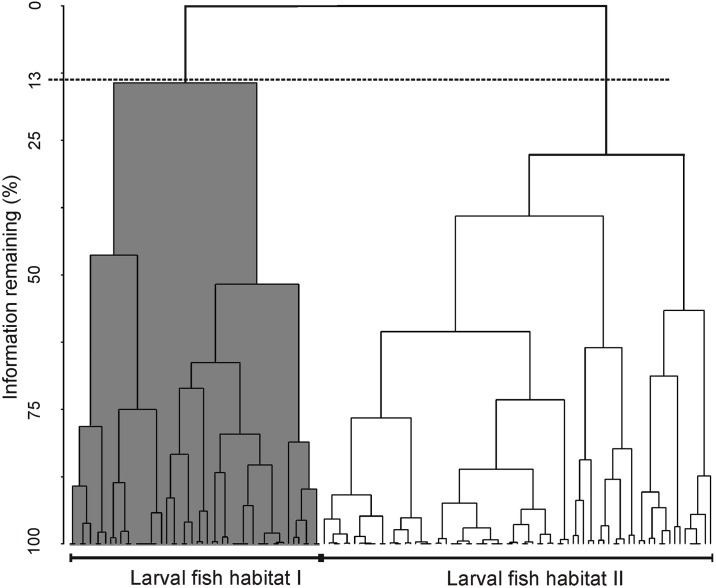
Dendrogram of larval fish samples defined by the Bray-Curtis dissimilarity derived from high geostrophic kinetic energy transects. Fish larvae data collected in samples from high energy zones in the southern Gulf of California.

In samples with high geostrophic kinetic energy flux affected by mesoscale eddies, total larval abundance again demonstrated no statistically significant differences between day and night (with 95% confidence level). The Bray-Curtis dissimilarity measure defined two larval fish habitats to a cut of 13% of the information remaining of the data set (Fig 11), which were significantly different (ANOSIM: *R* = -0.23, with 95% confidence level; View Eq (3)). In contrast with the low energy data set, the negative value in ANOSIM showed great variability within the habitats. The first habitat clustered most samples of the cyclonic eddy 2010 (Grey shaded in Fig 11); and the second habitat, clustered mainly samples from the anticyclonic eddy 2007 and the cyclonic eddy 2011 (Unshaded in Fig 11).

The first larval fish habitat, i.e. the cyclonic eddy 2010 Line H2, had the lowest larval average abundance of the study (25 larvae/10 m^2^) and was formed by 50 samples (Table 5). The dominant species were *V*. *lucetia* (surface affinity) and *D*. *laternatus* (depth affinity), which contributed with ~ 69% and 25% respectively. Both species were relatively abundant throughout the water column tending to avoid stations with the highest geostrophic kinetic energy (Fig 7c and 7d).

**Table 5:** Olmstead–Tukey test, One-way ANOSIM (View Eq (3)) and SIMPER analyses between larval fish habitats (LFH) classified according to the Bray-Curtis measured in highest geostrophic kinetic energetic zone.

Habitat	LFH I				LFH II			
Number of samples	50				77			
Mean zooplankton biomass (mL/1000m^3^)	467				206			
Mean larval abundance (larvae/10m^2^)	25				33			
Taxa	H	X	%F	%S	H	X	%F	%S
Vinciguerria lucetia	D	34	88	69	D	47	72	26
Diogenichthys laternatus	D	29	63	25	R	24	49	10
Benthosema panamense	R	18	21	4	D	29	80	36
Triphoturus mexicanus	R	7	44	2	D	30	75	27

R = -0.23, value in ANOSIM (Analysis of similarities); SIMPER, Similarity percentages.

H, hierarchy; X, mean abundance; %F, percentage of occurrence; %S, Similarity percentage (%Contribution); D, dominant; C, constant; F, frequent; R, rare.

In the second larval fish habitat, i.e. the cyclonic eddy 2011 and the anticyclonic eddy 2007; Lines H1 and H3 respectively, the larval average abundance was only slightly higher at 33 larvae/10 m^2^ in 77 samples. Even though *V*. *lucetia* larvae were dominant as in the first habitat, *B*. *panamense* and *T*. *mexicanus* larvae were also important here, marking the difference with the first habitat. The contribution of each of the three species was > 26% (Table 5). *V*. *lucetia* (Figs 6c and 8c) was distributed throughout the sampled water column, as in the first habitat. Similarly *T*. *mexicanus* larvae (Figs 6d and 8d) and *B*. *panamense* larvae (not shown) were located at all depths sampled, but tended to avoid stations with the highest geostrophic kinetic energy, and *T*. *mexicanus* showed its highest abundance in the first 100 m depth.

**Fig 12:**
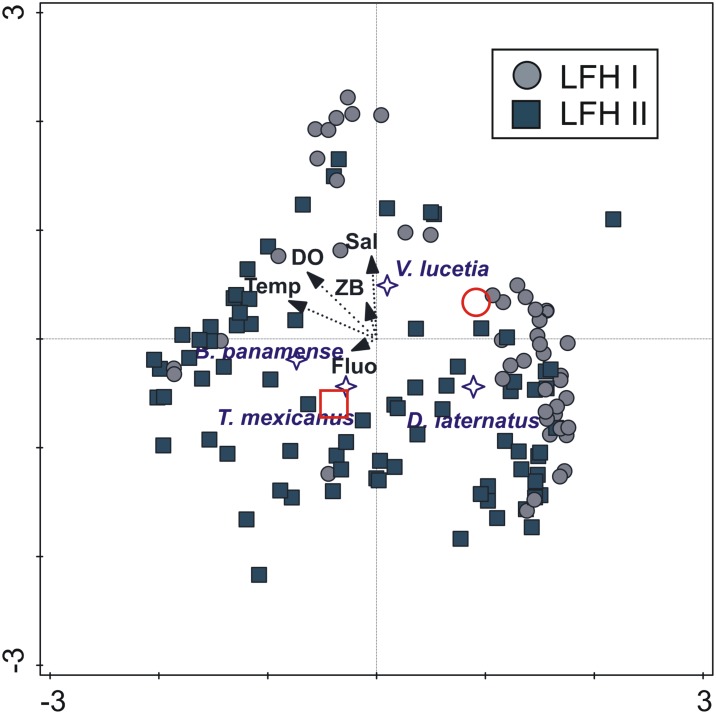
Triplot based on a Canonical Correspondence Analysis (CCA) ordination diagram from high geostrophic kinetic energy flux transects. Biological samples (squares and circles), larval fish habitat centroids (red symbols), species centroids (stars) and environmental data (arrows); first axis is horizontal, second axis vertical, collected in transects with high energy zones in southern of Gulf of California. Sal: Absolute salinity; ZB: zooplanktonic displacement volume; Fluo: fluorescence; Temp: Conservative temperature; DO: dissolved oxygen. LFH: Larval fish habitat.

The definitions of these two larval fish habitats were also identified by the CCA (Fig 12), with a relativity high relationship between variables (Pearson correlation 0.67) (Table 4). The eigenvalues of axis 1 (horizontal) and axis 2 (vertical) were 0.21 and 0.15, respectively; the eigenvalue of the axis 3 (not displayed) was 0.01. The most samples of the first larval fish habitat (cyclonic eddy 2010) corresponded to negative levels of temperature and dissolved oxygen and intermediate salinity, associating mainly with high larval abundance of *D*. *laternatus* and *V*. *lucetia*. On the other hand, most samples of the second larval fish habitat (cyclonic eddy 2011 and anticyclonic eddy 2007) were corresponded to positive values of temperature, fluorescence and dissolved oxygen (with a correlation of 51.7%, 46% and 28% respectively) and associated with high larval abundance of *T*. *mexicanus* and *B*. *panamense*.

## Discussion

This study examines the relation of geostrophic kinetic energy levels on the distribution of mesopelagic fish larvae (*V*. *lucetia*, *D*. *laternatus*, *B*. *panamense* and *T*. *mexicanus*) during the period of year with stronger stratification. Geostrophic kinetic energy-rich areas associated with mesoscale eddies were compared with energy-poor areas where the structure of the water column is more stable. Evidence was found of statistical relationships between larval fish abundance, their three-dimensional distribution, and the structure of the water column. Most previous studies (e.g. [61,34,62]) suggested relationships qualitatively, but did not make a statistical comparison of geostrophic kinetic energy-rich and energy-poor areas.

Because most of the samples were in the summer and early fall, the water column had relatively high values of the potential energy anomaly in all transects analyzed, as expected from previous work [63]. The present study considered a prior classification of the transects analyzed: *i)* those with little evidence of mesoscale activity, where the geostrophic kinetic energy was low (< 10 J/m^3^), with generally horizontal isotherms and isopycnals and geostrophic velocities < 0.1 m/s; and *ii*) transects with evident mesoscale activity exemplified by high geostrophic kinetic energy > 12 J/m^3^, and the presence of cyclonic and anticyclonic eddies, where the geostrophic velocities were > 0.1 m/s. In the first case (energy-poor areas), two larval fish habitats (with 95% confidence level) were statistically defined (Fig 9). One of them was dominated by larvae of *V*. *lucetia* and *B*. *panamense* located mostly in and above the pycnocline (typically located ~ 40 m depth); while the second habitat was characterized by larvae of *D*. *laternatus* mainly located in and below the pycnocline (Figs 2–5). This result coincides with previous qualitative observations of their vertical distribution (e.g. [31,34]). The statistical corroboration provided by the present study suggests that the *V*. *lucetia* and *B*. *panamense* larvae tended to congregate in the surface layer, while the *D*. *laternatus* larvae concentrated in the subsurface layer. The opposed depth distributions of the larvae of these mesopelagic species may imply different physiological adaptations to the environment, such as *V*. *lucetia* and *B*. *panamense* larvae preferring warm and productive water (> 20°C and > 1.5 mg/m^3^), while *D*. *laternatus* tolerates hypoxic (< 1 mL/L) conditions, as has been suggested in previous studies [29,64]; probably being an adaptive advantage of predator avoidance.

Despite this separation of larval fish habitats, all mesopelagic larvae coincided in the pycnocline, where chlorophyll maximums have been recorded in stratified conditions in different oceans [65]. Lasker *et al*. (1975) [65] related the presence of the chlorophyll maximum layer with the successful first feeding of larvae of epipelagic species (anchovy larvae). It is possible that the pycnocline and chlorophyll maximum layer also play an important role in the larval feeding of mesopelagic species, indicated in this study by their high abundance and frequency in that layer. Larvae associated with the “Surface larval fish habitat” migrate mainly in the mixing layer, between the chlorophyll maximum and surface, and in contrast, the species associated to the “Subsurface larval fish habitat”, migrate mainly between the deep layer and chlorophyll maximum. In both cases, the vertical migrations were confined to the sampled layer, which explain why there were no significant day-night differences in abundance.

Exceptions to these larval patterns were recorded in August 2005, when despite low geostrophic kinetic energy levels, the larvae of *B*. *panamense* and *V*. *lucetia* were distributed through the entire water column sampled, and no larvae of *D*. *laternatus* were found (Fig 2d). This circumstance was associated with positive anomalies of temperature corresponding to an El Niño event (http://www.esrl.noaa.gov/psd/enso/mei/) that apparently increased spawning of *B*. *panamense* and reduced that of *D*. *laternatus*. Although there is no literature specific to this issue, these changes in fish larvae abundance in August 2005 have been noted incidentally [66].

Two larval fish habitats were also statistically defined (with 95% confidence level) in sampling stations from geostrophic kinetic energy-rich areas, where mesoscale eddies were detected (Fig 11). In both habitats, the larval abundance of the dominant species was lower than in areas with low energy, and the larvae were more spread throughout the water column. One of the habitats was formed by samples from a cyclonic eddy (July 2010), where the geostrophic kinetic energy was relativity high, and *V*. *lucetia* and *D*. *laternatus* larvae had the lowest larval abundance of the study (Fig 7). The second habitat clustered mainly samples from transects influenced by the other cyclonic eddy (July 2011) and the anticyclonic eddy (October 2007), where the highest geostrophic kinetic energy of the study (> 12 J/m^3^) was recorded. In this last cluster, although *V*. *lucetia* was the dominant species, *B*. *panamense* and *T*. *mexicanus* were also important, in contrast to the cyclonic eddy (July 2010). Therefore the separation of the two clusters from geostrophic kinetic energy-rich areas corresponded more to changes in larval fish abundance, product of the intensity of spawning of each species, than to possible effects of the eddy rotation on the larval distribution.

The rotation of the eddy, regardless of its direction, might generate mixing by convergence and divergence, resulting in less stable conditions that favor both larval survival and subsequent recruitment, but vertical dispersal will decrease the availability of food for larvae, as mentioned Lasker *et al*. [65] for sardine larvae in the California Current.

The effect of the eddies on the zooplankton organisms is complex because eddies evolve over time as a result of processes such as diffusion and interaction with the wind [9,67]. Moreover, submesoscale processes like ageostrophic secondary circulation and mixing can modulate the plankton community distribution/structure through localized vertical fluxes at the eddy periphery [67–70]. Therefore more detailed observations and modelling will be required to fully understand the interaction of the zooplankton organisms with the eddy dynamics.

The physical phenomena that affect zooplankton occur at many scales simultaneously and may have synergistic effects that are difficult to measure [5,71–73]. It is considered that the mixing and convergence/divergence processes that occur in eddies are important in fertilization of surface waters of the ocean, and thus the subsequent enrichment of food webs [74]. However the results obtained in this work, in spite of the low resolution sampling, indicate that for primary consumers of the food chain, e.g., the mesopelagic fish larvae, the eddies may not provide the optimal conditions for larval development. Holliday *et al*. [75], Nieto *et al*. [13] and Song *et al*. [76], with different arguments, have suggested that the possibility of eddies not always being favorable for the development of fish eggs and larvae should be pursued in future multidisciplinary studies. It may be, however, that stratification of the water column is one of the most significant factors in the formation and maintenance of habitats of some zooplanktonic organisms [77], including the mesopelagic fish larvae.

The results of this work in the southern Gulf of California, based on transects influenced by mesoscale eddies (geostrophic kinetic energy-rich areas) and on transects with weak mesoscale activity (energy-poor areas), during the seasons of strongest stratification, suggested that the level of the geostrophic kinetic energy affects the abundance and three-dimensional distribution of the mesopelagic fish larvae. As the highest larval abundance was correlated with low geostrophic kinetic energy flux, and the larval congregation was higher in samples from low-energy areas, it appears that geostrophic kinetic energy-rich areas do not provide the best conditions for mesopelagic larval development. Although there are relatively few observations with which to generalize, and more extensive statistical analyses are desirable, it is tempting to speculate that this result could well apply to other regions.

## Supporting Information

S1 Table A Number of fish larvae in lines with low geostrophic kinetic energy flux. B Table Average of environmental variables in lines with low geostrophic kinetic energy flux.
(DOCX)

S2 Table A Number of fish larvae in lines with high geostrophic kinetic energy flux. B Table Average of environmental variables in lines with high geostrophic kinetic energy flux.
(DOCX)
