# Supplementary material for: Effects of Geostrophic Kinetic Energy on the Distribution of Mesopelagic Fish Larvae in the Southern Gulf of California in Summer/Fall Stratified Seasons
Source: PLoS One. 2016 Oct 19;11(10):e0164900. doi: 10.1371/journal.pone.0164900 (PMC5070878; doi:10.1371/journal.pone.0164900)
Supplement: S1 Table — A Number of fish larvae in lines with low geostrophic kinetic energy flux. B Table Average of environmental variables in lines with low geostrophic kinetic energy flux. (DOCX) [file pone.0164900.s001.docx]

S1A Table Number of fish larvae in lines with low energy anomalies

| Station | Strata | *Benthosema panamense* | *Diogenichthys laternatus* | *Triphoturus mexicanus* | *Vinciguerria lucetia* |
| --- | --- | --- | --- | --- | --- |
| A1 | 0-50 | 2 | 0 | 9 | 0 |
| A2 | 0-50 | 7 | 0 | 3 | 0 |
| A3 | 0-50 | 29 | 0 | 6 | 0 |
| A3 | 50-100 | 29 | 0 | 3 | 0 |
| A3 | 150-200 | 3 | 2 | 4 | 0 |
| B1 | 0-50 | 79 | 0 | 1 | 0 |
| B1 | 50-100 | 20 | 0 | 1 | 0 |
| B2 | 0-50 | 364 | 0 | 0 | 1 |
| B2 | 50-100 | 4 | 0 | 3 | 1 |
| B2 | 50-100 | 11 | 0 | 0 | 0 |
| B2 | 100-150 | 15 | 0 | 0 | 0 |
| B3 | 0-50 | 79 | 0 | 0 | 1 |
| B3 | 0-50 | 104 | 0 | 1 | 0 |
| B3 | 0-50 | 126 | 0 | 2 | 0 |
| B3 | 50-100 | 1 | 0 | 0 | 0 |
| B3 | 100-150 | 36 | 0 | 0 | 0 |
| B4 | 0-50 | 15 | 0 | 0 | 0 |
| B4 | 50-100 | 60 | 0 | 0 | 0 |
| B4 | 150-200 | 52 | 0 | 0 | 0 |
| B5 | 0-50 | 274 | 0 | 0 | 0 |
| B5 | 50-100 | 19 | 0 | 0 | 0 |
| B5 | 100-150 | 35 | 0 | 0 | 0 |
| B5 | 150-200 | 22 | 0 | 0 | 0 |
| B6 | 0-50 | 12 | 0 | 0 | 0 |
| A02 | 0-15 | 3 | 0 | 0 | 71 |
| A02 | 15-30 | 0 | 0 | 3 | 18 |
| A02 | 30-45 | 8 | 17 | 31 | 706 |
| A02 | 50-100 | 2 | 17 | 135 | 55 |
| A02 | 100-150 | 0 | 15 | 1 | 3 |
| A02 | 150-200 | 0 | 6 | 1 | 2 |
| A03 | 0-15 | 1 | 1 | 0 | 28 |
| A03 | 15-30 | 1 | 5 | 12 | 130 |
| A03 | 30-45 | 0 | 4 | 14 | 69 |
| A03 | 50-100 | 0 | 5 | 3 | 9 |
| A03 | 100-150 | 1 | 16 | 1 | 10 |
| A03 | 150-200 | 0 | 1 | 0 | 1 |
| A04 | 0-15 | 4 | 0 | 0 | 13 |
| A04 | 15-30 | 17 | 0 | 2 | 74 |
| A04 | 30-45 | 1 | 1 | 5 | 41 |
| A04 | 50-100 | 0 | 0 | 0 | 0 |
| A04 | 100-150 | 0 | 43 | 2 | 6 |
| A04 | 150-200 | 0 | 0 | 0 | 2 |
| A05 | 0-15 | 0 | 0 | 0 | 3 |
| A05 | 15-30 | 2 | 1 | 3 | 20 |
| A05 | 30-45 | 0 | 4 | 0 | 114 |
| A05 | 50-100 | 0 | 1 | 0 | 1 |
| A05 | 100-150 | 0 | 8 | 0 | 1 |
| A05 | 150-200 | 0 | 0 | 0 | 0 |
| B01 | 0-15 | 9 | 0 | 4 | 32 |
| B01 | 15-30 | 0 | 0 | 42 | 10 |
| B01 | 30-45 | 0 | 11 | 15 | 0 |
| B01 | 50-100 | 1 | 26 | 7 | 2 |
| B01 | 100-150 | 0 | 2 | 6 | 2 |
| B01 | 150-200 | 0 | 2 | 4 | 0 |
| B02 | 0-15 | 0 | 0 | 1 | 6 |
| B02 | 15-30 | 0 | 0 | 2 | 5 |
| B02 | 30-45 | 0 | 0 | 1 | 0 |
| B02 | 50-100 | 1 | 0 | 0 | 2 |
| B02 | 100-150 | 0 | 2 | 0 | 0 |
| B02 | 150-200 | 0 | 2 | 2 | 1 |
| B03 | 0-15 | 1 | 1 | 11 | 166 |
| B03 | 15-30 | 0 | 0 | 0 | 3 |
| B03 | 30-45 | 1 | 6 | 34 | 100 |
| B03 | 50-100 | 0 | 0 | 0 | 1 |
| B03 | 100-150 | 0 | 2 | 2 | 0 |
| B03 | 150-200 | 0 | 2 | 0 | 1 |
| B04 | 0-15 | 3 | 33 | 11 | 62 |
| B04 | 15-30 | 5 | 0 | 0 | 15 |
| B04 | 30-45 | 11 | 4 | 13 | 99 |
| B04 | 50-100 | 4 | 8 | 2 | 33 |
| B04 | 100-150 | 0 | 4 | 0 | 2 |
| B04 | 150-200 | 0 | 1 | 0 | 0 |
| B05 | 0-15 | 61 | 0 | 0 | 30 |
| B05 | 15-30 | 12 | 0 | 0 | 19 |
| B05 | 30-45 | 20 | 12 | 0 | 52 |
| B05 | 50-100 | 19 | 28 | 3 | 59 |
| B05 | 100-150 | 3 | 6 | 1 | 7 |
| B05 | 150-200 | 0 | 37 | 2 | 9 |
| B06 | 0-15 | 0 | 0 | 0 | 0 |
| B06 | 15-30 | 0 | 0 | 0 | 5 |
| B06 | 30-45 | 1 | 0 | 0 | 1 |
| B06 | 50-100 | 11 | 0 | 0 | 1 |
| B06 | 100-150 | 1 | 0 | 0 | 0 |
| B06 | 150-200 | 0 | 1 | 0 | 0 |
| B07 | 0-15 | 0 | 0 | 0 | 1 |
| B07 | 15-30 | 0 | 0 | 0 | 0 |
| B07 | 30-45 | 0 | 0 | 0 | 0 |
| B07 | 50-100 | 31 | 4 | 0 | 2 |
| B07 | 100-150 | 1 | 8 | 0 | 0 |
| B07 | 150-200 | 4 | 0 | 0 | 0 |
| B08 | 0-15 | 0 | 1 | 1 | 21 |
| B08 | 15-30 | 0 | 0 | 0 | 4 |
| B08 | 30-45 | 8 | 1 | 8 | 95 |
| B08 | 50-100 | 7 | 13 | 5 | 109 |
| B08 | 100-150 | 0 | 9 | 1 | 1 |
| B08 | 150-200 | 0 | 1 | 0 | 2 |
| B09 | 0-15 | 108 | 0 | 0 | 52 |
| B09 | 15-30 | 149 | 0 | 2 | 174 |
| B09 | 30-45 | 24 | 0 | 0 | 25 |
| B09 | 50-100 | 0 | 0 | 0 | 0 |
| B09 | 100-150 | 0 | 23 | 2 | 29 |
| B09 | 150-200 | 0 | 31 | 0 | 1 |
| B10 | 0-15 | 53 | 2 | 0 | 107 |
| B10 | 15-30 | 5 | 0 | 0 | 0 |
| B10 | 30-45 | 0 | 1 | 0 | 21 |
| B10 | 50-100 | 0 | 1 | 0 | 13 |
| B10 | 100-150 | 0 | 5 | 1 | 2 |
| B10 | 150-200 | 0 | 0 | 1 | 0 |
| A01 | 0-17 | 3 | 0 | 0 | 2 |
|  | 17-34 | 7 | 0 | 14 | 17 |
|  | 34-51 | 5 | 3 | 35 | 10 |
|  | 50-100 | 2 | 1 | 14 | 10 |
| A02 | 0-17 | 3 | 0 | 0 | 5 |
|  | 17-34 | 13 | 0 | 46 | 26 |
|  | 34-51 | 2 | 4 | 36 | 10 |
|  | 50-100 | 0 | 0 | 7 | 2 |
|  | 100-150 | 1 | 7 | 3 | 1 |
|  | 150-200 | 0 | 1 | 2 | 0 |
| A03 | 0-17 | 2 | 0 | 11 | 2 |
|  | 17-34 | 1 | 0 | 13 | 3 |
|  | 34-51 | 2 | 0 | 4 | 2 |
|  | 100-150 | 1 | 0 | 2 | 0 |
|  | 150-200 | 0 | 0 | 2 | 0 |
| A14 | 0-17 | 5 | 0 | 17 | 11 |
|  | 17-34 | 10 | 0 | 0 | 5 |
|  | 34-51 | 3 | 13 | 10 | 17 |
|  | 50-100 | 0 | 5 | 1 | 1 |
|  | 100-150 | 0 | 4 | 0 | 3 |
|  | 150-200 | 0 | 4 | 1 | 2 |
| A15 | 0-17 | 0 | 0 | 0 | 0 |
|  | 17-34 | 0 | 1 | 8 | 2 |
|  | 34-51 | 2 | 1 | 3 | 2 |
|  | 50-100 | 0 | 2 | 3 | 0 |
|  | 100-150 | 0 | 0 | 0 | 0 |
|  | 150-200 | 0 | 0 | 0 | 0 |
| A16 | 0-17 | 10 | 0 | 7 | 4 |
|  | 17-34 | 19 | 1 | 10 | 10 |
|  | 34-51 | 10 | 13 | 37 | 19 |
|  | 50-100 | 0 | 2 | 0 | 2 |
|  | 100-150 | 0 | 0 | 0 | 1 |
| A17 | 0-17 | 5 | 0 | 0 | 4 |
|  | 17-34 | 19 | 0 | 31 | 63 |
|  | 34-51 | 26 | 8 | 41 | 30 |
|  | 50-100 | 6 | 13 | 13 | 15 |
|  | 100-150 | 2 | 3 | 12 | 6 |
|  | 150-200 | 0 | 0 | 5 | 2 |
| A18 | 0-17 | 1 | 0 | 0 | 0 |
|  | 17-34 | 20 | 0 | 3 | 23 |
|  | 34-51 | 24 | 0 | 10 | 7 |
|  | 50-100 | 10 | 0 | 3 | 3 |
|  | 100-150 | 2 | 5 | 6 | 3 |
|  | 150-200 | 0 | 7 | 5 | 0 |
| A19 | 0-17 | 0 | 0 | 0 | 0 |
|  | 17-34 | 8 | 0 | 5 | 6 |
|  | 34-51 | 50 | 11 | 11 | 31 |
|  | 50-100 | 12 | 20 | 25 | 45 |
|  | 100-150 | 0 | 3 | 1 | 0 |
|  | 150-200 | 1 | 2 | 3 | 1 |
| C02 | 0-17 | 5 | 0 | 1 | 1 |
|  | 17-34 | 8 | 0 | 19 | 22 |
|  | 50-100 | 0 | 14 | 19 | 1 |
|  | 100-150 | 1 | 8 | 4 | 3 |
|  | 150-200 | 1 | 12 | 20 | 2 |
| C05 | 0-17 | 6 | 0 | 0 | 1 |
|  | 17-34 | 13 | 22 | 12 | 14 |
|  | 34-51 | 0 | 0 | 3 | 0 |
|  | 50-100 | 0 | 4 | 1 | 0 |
|  | 100-150 | 0 | 2 | 2 | 0 |
|  | 150-200 | 2 | 3 | 1 | 3 |
| C06 | 0-17 | 6 | 2 | 3 | 10 |
|  | 17-34 | 1 | 1 | 0 | 3 |
|  | 34-51 | 10 | 8 | 9 | 30 |
|  | 50-100 | 0 | 0 | 0 | 2 |
|  | 100-150 | 0 | 3 | 0 | 1 |
|  | 150-200 | 0 | 2 | 0 | 6 |
| C08 | 0-17 | 1 | 0 | 0 | 1 |
|  | 17-34 | 4 | 3 | 0 | 18 |
|  | 34-51 | 3 | 3 | 3 | 5 |
|  | 50-100 | 0 | 11 | 1 | 2 |
|  | 100-150 | 0 | 4 | 1 | 2 |
| C09 | 0-17 | 2 | 0 | 0 | 3 |
|  | 17-34 | 14 | 4 | 14 | 66 |
|  | 34-51 | 0 | 5 | 3 | 22 |
|  | 50-100 | 1 | 6 | 0 | 7 |
|  | 100-150 | 3 | 32 | 8 | 39 |
|  | 150-200 | 0 | 29 | 1 | 10 |
| C11 | 0-17 | 2 | 0 | 0 | 5 |
|  | 17-34 | 2 | 3 | 9 | 67 |
|  | 50-100 | 1 | 9 | 44 | 160 |
|  | 100-150 | 0 | 1 | 5 | 11 |
|  | 150-200 | 0 | 2 | 2 | 11 |
| C12 | 0-17 | 2 | 0 | 0 | 1 |
|  | 17-34 | 12 | 0 | 0 | 11 |
|  | 34-51 | 36 | 20 | 35 | 219 |
|  | 50-100 | 10 | 50 | 51 | 330 |
|  | 100-150 | 4 | 3 | 0 | 10 |
|  | 150-200 | 2 | 5 | 4 | 3 |
| C13 | 0-17 | 1 | 0 | 0 | 0 |
|  | 17-34 | 5 | 2 | 1 | 3 |
|  | 34-51 | 9 | 1 | 1 | 2 |
|  | 50-100 | 0 | 4 | 0 | 2 |
|  | 100-150 | 0 | 0 | 0 | 0 |
|  | 150-200 | 0 | 2 | 0 | 1 |
| C17 | 0-17 | 34 | 0 | 0 | 7 |
|  | 17-34 | 14 | 0 | 0 | 4 |
|  | 34-51 | 4 | 1 | 0 | 0 |
|  | 50-100 | 1 | 1 | 1 | 0 |
|  | 100-150 | 1 | 4 | 1 | 0 |
|  | 150-200 | 0 | 5 | 0 | 0 |
| C18 | 0-17 | 10 | 0 | 2 | 0 |
|  | 17-34 | 18 | 0 | 2 | 4 |
|  | 34-51 | 17 | 0 | 6 | 9 |
|  | 50-100 | 6 | 1 | 10 | 4 |
|  | 100-150 | 2 | 0 | 4 | 0 |
|  | 150-200 | 0 | 1 | 2 | 0 |
| C20 | 0-17 | 1 | 0 | 7 | 1 |
|  | 17-34 | 16 | 0 | 6 | 7 |
|  | 34-51 | 3 | 0 | 4 | 0 |
|  | 50-100 | 0 | 1 | 0 | 0 |
|  | 100-150 | 0 | 3 | 0 | 0 |
|  | 150-200 | 0 | 2 | 0 | 1 |
| C21 | 0-17 | 7 | 0 | 1 | 1 |
|  | 17-34 | 155 | 0 | 16 | 0 |
|  | 34-51 | 180 | 3 | 24 | 1 |
|  | 50-100 | 22 | 2 | 14 | 1 |
|  | 100-150 | 2 | 0 | 0 | 0 |
|  | 150-200 | 0 | 1 | 1 | 0 |
| 1 | 0-50 | 19 | 0 | 2 | 6 |
|  | 50-100 | 11 | 2 | 3 | 3 |
|  | 150-200 | 1 | 3 | 2 | 0 |
| 2 | 0-50 | 5 | 0 | 2 | 0 |
|  | 50-100 | 13 | 0 | 1 | 3 |
|  | 100-150 | 2 | 1 | 0 | 0 |
|  | 150-200 | 1 | 5 | 0 | 0 |
| 3 | 0-50 | 3 | 2 | 1 | 4 |
|  | 50-100 | 5 | 8 | 1 | 2 |
|  | 100-150 | 2 | 0 | 0 | 0 |
|  | 150-200 | 0 | 4 | 0 | 0 |
| 4 | 0-50 | 2 | 0 | 2 | 12 |
|  | 50-100 | 0 | 0 | 1 | 6 |
|  | 100-150 | 3 | 0 | 3 | 12 |
|  | 150-200 | 3 | 0 | 0 | 5 |
| 5 | 0-50 | 5 | 0 | 4 | 12 |
|  | 50-100 | 4 | 2 | 2 | 4 |
|  | 100-150 | 7 | 10 | 10 | 16 |
|  | 150-200 | 2 | 0 | 0 | 1 |
| 6 | 0-50 | 5 | 0 | 2 | 15 |
|  | 50-100 | 11 | 0 | 3 | 9 |
|  | 100-150 | 0 | 0 | 0 | 1 |
|  | 150-200 | 1 | 4 | 1 | 3 |
| 7 | 0-50 | 2 | 0 | 0 | 4 |
|  | 50-100 | 3 | 0 | 5 | 6 |
|  | 100-150 | 1 | 1 | 6 | 9 |
|  | 150-200 | 8 | 2 | 11 | 6 |
| 8 | 0-50 | 0 | 0 | 1 | 3 |
|  | 50-100 | 0 | 1 | 2 | 0 |
|  | 100-150 | 1 | 1 | 0 | 0 |
|  | 150-200 | 0 | 1 | 0 | 0 |
| 9 | 0-50 | 14 | 3 | 1 | 0 |
|  | 50-100 | 12 | 2 | 0 | 0 |
|  | 100-150 | 2 | 1 | 0 | 0 |
|  | 150-200 | 0 | 7 | 1 | 0 |
| 10 | 0-50 | 9 | 0 | 5 | 7 |
|  | 50-100 | 6 | 1 | 3 | 2 |
|  | 100-150 | 6 | 1 | 7 | 12 |
|  | 150-200 | 0 | 0 | 0 | 1 |
| 11 | 0-50 | 32 | 0 | 26 | 7 |
|  | 50-100 | 10 | 1 | 19 | 1 |
|  | 100-150 | 0 | 0 | 2 | 0 |
|  | 150-200 | 7 | 2 | 9 | 3 |
| 12 | 0-50 | 6 | 0 | 12 | 15 |
|  | 50-100 | 0 | 1 | 9 | 0 |
|  | 100-150 | 10 | 0 | 8 | 29 |
|  | 150-200 | 0 | 0 | 3 | 1 |
| 13 | 0-50 | 16 | 0 | 3 | 21 |
|  | 50-100 | 1 | 10 | 3 | 7 |
|  | 100-150 | 1 | 0 | 0 | 2 |
|  | 150-200 | 7 | 2 | 1 | 1 |
| A1 | A01(CM) | 0 | 12 | 2 | 350 |
| A4 | A04(CM) | 180 | 0 | 0 | 36 |
| A7 | A07(CM) | 0 | 3 | 0 | 190 |
| A10 | A10(CM) | 0 | 4 | 0 | 369 |
| A13 | A13(CM) | 15 | 0 | 0 | 143 |
| A16 | A16(CM) | 0 | 0 | 0 | 76 |
| B1 | B01(CM) | 0 | 200 | 25 | 480 |
| B4 | B04(CM) | 0 | 2 | 0 | 73 |
| B7 | B07(CM) | 25 | 0 | 0 | 1100 |
| B10 | B10(CM) | 55 | 21 | 0 | 3576 |
| B13B | B13(CM) | 20 | 0 | 0 | 712 |
| E11 | E11(CM) | 0 | 0 | 45 | 186 |
| E13 | E13(CM) | 0 | 0 | 6 | 282 |
| E15 | E15(CM) | 0 | 0 | 43 | 671 |
| E17 | E17(CM) | 0 | 0 | 9 | 147 |
| E19 | E19(CM) | 72 | 9 | 0 | 974 |
| E21 | E21(CM) | 0 | 0 | 0 | 52 |
| E23 | E23(CM) | 0 | 26 | 0 | 356 |
| E25 | E25(CM) | 40 | 0 | 0 | 536 |
| E27 | E27(CM) | 11 | 0 | 0 | 69 |
| E29 | E29(CM) | 190 | 0 | 0 | 195 |
| E31 | E31(CM) | 22 | 0 | 0 | 17 |
| E33 | E33(CM) | 69 | 0 | 0 | 54 |
| E35 | E35(CM) | 0 | 0 | 0 | 23 |
| E37 | E37(CM) | 0 | 0 | 0 | 0 |
| C1 | C01(CM) | 0 | 0 | 0 | 65 |
| C3 | C03(CM) | 0 | 0 | 0 | 682 |
| C5 | C05(CM) | 13 | 0 | 0 | 245 |
| C7 | C07(CM) | 0 | 27 | 0 | 168 |
| C9 | C09(CM) | 2 | 0 | 0 | 165 |
| C11 | C11(CM) | 13 | 23 | 0 | 148 |
| C13 | C13(CM) | 4 | 0 | 0 | 114 |
| C15 | C15(CM) | 0 | 0 | 0 | 508 |
| C17 | C17(CM) | 0 | 6 | 0 | 1250 |
| A1 | A01(T) | 0 | 69 | 0 | 1071 |
| A4 | A04(T) | 185 | 13 | 8 | 374 |
| A7 | A07(T) | 0 | 64 | 0 | 19 |
| A10 | A10(T) | 17 | 79 | 4 | 1910 |
| A13 | A13(T) | 12 | 27 | 0 | 42 |
| B1 | B01(T) | 0 | 91 | 0 | 123 |
| B4 | B04(T) | 13 | 280 | 7 | 93 |
| B7 | B07(T) | 0 | 490 | 0 | 3212 |
| B10 | B10(T) | 0 | 784 | 29 | 21557 |
| B13 | B13(T) | 0 | 86 | 14 | 839 |
| E11 | E11(T) | 0 | 2 | 20 | 240 |
| E13 | E13(T) | 8 | 297 | 12 | 1075 |
| E15 | E15(T) | 0 | 9 | 0 | 101 |
| E17 | E17(T) | 0 | 800 | 43 | 4076 |
| E19 | E10(T) | 103 | 0 | 0 | 424 |
| E21 | E21(T) | 0 | 5 | 3 | 428 |
| E23 | E23(T) | 0 | 206 | 0 | 1001 |
| E25 | E25(T) | 0 | 40 | 0 | 2267 |
| E27 | E27(T) | 17 | 6 | 0 | 318 |
| E29 | E29(T) | 229 | 0 | 0 | 103 |
| E31 | E31(T) | 206 | 0 | 0 | 175 |
| E33 | E33(T) | 449 | 0 | 0 | 145 |
| E35 | E35(T) | 0 | 0 | 0 | 4 |
| E37 | E37(T) | 0 | 0 | 0 | 0 |
| C1 | C01(T) | 0 | 0 | 0 | 67 |
| C3 | C03(T) | 29 | 55 | 3 | 3700 |
| C5 | C05(T) | 0 | 48 | 6 | 0 |
| C7 | C07(T) | 45 | 0 | 0 | 909 |
| C9 | C09(T) | 120 | 0 | 0 | 869 |
| C11 | C11(T) | 14 | 77 | 0 | 11 |
| C13 | C13(T) | 14 | 0 | 0 | 223 |
| C15 | C15(T) | 8 | 25 | 0 | 1381 |
| C17 | C17(T) | 0 | 218 | 0 | 259 |
| A1 | A01(ZMO) | 0 | 27 | 0 | 0 |
| A7 | A07(ZMO) | 0 | 9 | 0 | 9 |
| A10 | A10(ZMO) | 0 | 20 | 0 | 11 |
| A13 | A13(ZMO) | 0 | 36 | 0 | 5 |
| B1 | B01(ZMO) | 0 | 17 | 0 | 2 |
| B4 | B04(ZMO) | 0 | 19 | 0 | 0 |
| B7 | B07(ZMO) | 0 | 167 | 0 | 47 |
| B10 | B10(ZMO) | 0 | 381 | 2 | 260 |
| B13 | B13(ZMO) | 0 | 44 | 0 | 22 |
| E11 | E11(ZMO) | 0 | 42 | 0 | 6 |
| E13 | E13(ZMO) | 0 | 105 | 0 | 13 |
| E15 | E15(ZMO) | 0 | 129 | 2 | 76 |
| E17 | E17(ZMO) | 0 | 187 | 1 | 69 |
| E19 | E19(ZMO) | 0 | 1 | 0 | 15 |
| E21 | E21(ZMO) | 57 | 18 | 0 | 550 |
| E23 | E23(ZMO) | 0 | 20 | 0 | 1 |
| E25 | E25(ZMO) | 0 | 17 | 0 | 302 |
| E27 | E27(ZMO) | 0 | 17 | 0 | 6 |
| E29 | E29(ZMO) | 0 | 5 | 0 | 2 |
| E31 | E31(ZMO) | 19 | 0 | 0 | 2 |
| E33 | E33(ZMO) | 60 | 0 | 0 | 36 |
| E35 | E35(ZMO) | 0 | 0 | 0 | 0 |
| E37 | E37(ZMO) | 0 | 0 | 0 | 0 |
| C1 | C01(ZMO) | 0 | 0 | 0 | 3 |
| C3 | C03(ZMO) | 2 | 39 | 1 | 344 |
| C5 | C05(ZMO) | 0 | 18 | 2 | 13 |
| C9 | C09(ZMO) | 0 | 13 | 0 | 6 |
| C11 | C11(ZMO) | 0 | 176 | 0 | 5 |
| C13 | C13(ZMO) | 0 | 53 | 0 | 176 |
| C15 | C15(ZMO) | 3 | 32 | 0 | 136 |
| C17 | C17(ZMO) | 0 | 43 | 0 | 13 |

S1B Table Average of environmental variables in lines with low energy anomalies

| Station | Strata | Temperature | Salinity | Dissolved oxygen | Fluorescence | Zooplankton displacement biomass |
| --- | --- | --- | --- | --- | --- | --- |
| A1 | 0-50 | 27 | 35 | 4 | 1 | 409 |
| A2 | 0-50 | 19 | 35 | 3 | 0 | 114 |
| A3 | 0-50 | 15 | 35 | 1 | 0 | 75 |
| A3 | 50-100 | 13 | 35 | 1 | 0 | 45 |
| A3 | 150-200 | 28 | 35 | 4 | 0 | 330 |
| B1 | 0-50 | 15 | 35 | 2 | 0 | 19 |
| B1 | 50-100 | 27 | 35 | 4 | 0 | 195 |
| B2 | 0-50 | 18 | 35 | 2 | 0 | 77 |
| B2 | 50-100 | 14 | 35 | 1 | 0 | 43 |
| B2 | 50-100 | 27 | 35 | 4 | 1 | 233 |
| B2 | 100-150 | 20 | 35 | 3 | 0 | 72 |
| B3 | 0-50 | 27 | 35 | 4 | 0 | 116 |
| B3 | 0-50 | 20 | 35 | 2 | 0 | 100 |
| B3 | 0-50 | 16 | 35 | 2 | 0 | 34 |
| B3 | 50-100 | 14 | 35 | 1 | 0 | 18 |
| B3 | 100-150 | 28 | 35 | 4 | 0 | 501 |
| B4 | 0-50 | 17 | 35 | 2 | 0 | 52 |
| B4 | 50-100 | 29 | 35 | 4 | 0 | 37 |
| B4 | 150-200 | 23 | 35 | 3 | 0 | 181 |
| B5 | 0-50 | 14 | 35 | 1 | 0 | 40 |
| B5 | 50-100 | 29 | 35 | 4 | 0 | 30 |
| B5 | 100-150 | 22 | 35 | 3 | 0 | 122 |
| B5 | 150-200 | 16 | 35 | 2 | 0 | 25 |
| B6 | 0-50 | 14 | 35 | 1 | 0 | 50 |
| A02 | 0-15 | 27 | 35 | 4 | 0 | 223 |
| A02 | 15-30 | 25 | 35 | 3 | 0 | 229 |
| A02 | 30-45 | 16 | 35 | 2 | 0 | 272 |
| A02 | 50-100 | 12 | 35 | 0 | 0 | 54 |
| A02 | 100-150 | 25 | 35 | 3 | 1 | 273 |
| A02 | 150-200 | 16 | 35 | 2 | 0 | 78 |
| A03 | 0-15 | 14 | 35 | 1 | 0 | 38 |
| A03 | 15-30 | 13 | 35 | 0 | 0 | 13 |
| A03 | 30-45 | 25 | 35 | 3 | 1 | 292 |
| A03 | 50-100 | 25 | 35 | 3 | 1 | 363 |
| A03 | 100-150 | 26 | 35 | 4 | 1 | 317 |
| A03 | 150-200 | 16 | 35 | 1 | 0 | 116 |
| A04 | 0-15 | 12 | 35 | 0 | 0 | 50 |
| A04 | 15-30 | 26 | 35 | 4 | 0 | 196 |
| A04 | 30-45 | 17 | 35 | 2 | 0 | 98 |
| A04 | 50-100 | 14 | 35 | 1 | 0 | 75 |
| A04 | 100-150 | 25 | 35 | 3 | 0 | 157 |
| A04 | 150-200 | 17 | 35 | 2 | 0 | 48 |
| A05 | 0-15 | 14 | 35 | 0 | 0 | 73 |
| A05 | 15-30 | 12 | 35 | 0 | 0 | 41 |
| A05 | 30-45 | 26 | 35 | 4 | 0 | 265 |
| A05 | 50-100 | 12 | 35 | 0 | 0 | 38 |
| A05 | 100-150 | 27 | 35 | 4 | 0 | 211 |
| A05 | 150-200 | 18 | 35 | 1 | 0 | 25 |
| B01 | 0-15 | 13 | 35 | 0 | 0 | 32 |
| B01 | 15-30 | 27 | 35 | 4 | 0 | 214 |
| B01 | 30-45 | 19 | 35 | 1 | 0 | 78 |
| B01 | 50-100 | 14 | 35 | 0 | 0 | 68 |
| B01 | 100-150 | 28 | 35 | 4 | 0 | 172 |
| B01 | 150-200 | 20 | 35 | 2 | 0 | 89 |
| B02 | 0-15 | 14 | 35 | 0 | 0 | 99 |
| B02 | 15-30 | 13 | 35 | 0 | 0 | 48 |
| B02 | 30-45 | 28 | 35 | 4 | 0 | 153 |
| B02 | 50-100 | 21 | 35 | 2 | 0 | 122 |
| B02 | 100-150 | 15 | 35 | 1 | 0 | 59 |
| B02 | 150-200 | 13 | 35 | 0 | 0 | 63 |
| B03 | 0-15 | 28 | 35 | 4 | 0 | 55 |
| B03 | 15-30 | 20 | 35 | 2 | 0 | 30 |
| B03 | 30-45 | 15 | 35 | 1 | 0 | 51 |
| B03 | 50-100 | 13 | 35 | 0 | 0 | 47 |
| B03 | 100-150 | 27 | 35 | 4 | 0 | 229 |
| B03 | 150-200 | 21 | 35 | 2 | 0 | 96 |
| B04 | 0-15 | 14 | 35 | 0 | 0 | 89 |
| B04 | 15-30 | 12 | 35 | 0 | 0 | 64 |
| B04 | 30-45 | 29 | 35 | 4 | 1 | 135 |
| B04 | 50-100 | 21 | 35 | 2 | 0 | 83 |
| B04 | 100-150 | 14 | 35 | 1 | 0 | 49 |
| B04 | 150-200 | 28 | 35 | 4 | 0 | 310 |
| B05 | 0-15 | 20 | 35 | 2 | 0 | 78 |
| B05 | 15-30 | 14 | 35 | 1 | 0 | 61 |
| B05 | 30-45 | 13 | 35 | 0 | 0 | 39 |
| B05 | 50-100 | 28 | 35 | 4 | 0 | 138 |
| B05 | 100-150 | 20 | 35 | 2 | 0 | 70 |
| B05 | 150-200 | 13 | 35 | 0 | 0 | 28 |
| B06 | 0-15 | 28 | 35 | 4 | 0 | 234 |
| B06 | 15-30 | 20 | 35 | 2 | 0 | 100 |
| B06 | 30-45 | 13 | 35 | 1 | 0 | 45 |
| B06 | 50-100 | 28 | 35 | 4 | 0 | 24 |
| B06 | 100-150 | 20 | 35 | 2 | 0 | 104 |
| B06 | 150-200 | 28 | 35 | 4 | 0 | 256 |
| B07 | 0-15 | 20 | 35 | 2 | 0 | 77 |
| B07 | 15-30 | 14 | 35 | 0 | 0 | 117 |
| B07 | 30-45 | 13 | 35 | 0 | 0 | 61 |
| B07 | 50-100 | 29 | 35 | 4 | 1 | 282 |
| B07 | 100-150 | 22 | 35 | 2 | 0 | 79 |
| B07 | 150-200 | 14 | 35 | 1 | 0 | 77 |
| B08 | 0-15 | 12 | 35 | 0 | 0 | 43 |
| B08 | 15-30 | 29 | 35 | 4 | 1 | 282 |
| B08 | 30-45 | 23 | 35 | 2 | 0 | 129 |
| B08 | 50-100 | 16 | 35 | 1 | 0 | 95 |
| B08 | 100-150 | 12 | 35 | 0 | 0 | 48 |
| B08 | 150-200 | 29 | 35 | 4 | 0 | 337 |
| B09 | 0-15 | 22 | 35 | 2 | 0 | 115 |
| B09 | 15-30 | 15 | 35 | 1 | 0 | 44 |
| B09 | 30-45 | 28 | 35 | 4 | 0 | 324 |
| B09 | 50-100 | 26 | 35 | 5 | 0 | 49 |
| B09 | 100-150 | 23 | 35 | 5 | 0 | 393 |
| B09 | 150-200 | 17 | 35 | 2 | 0 | 490 |
| B10 | 0-15 | 15 | 35 | 2 | 0 | 58 |
| B10 | 15-30 | 13 | 35 | 1 | 0 | 91 |
| B10 | 30-45 | 28 | 35 | 4 | 0 | 295 |
| B10 | 50-100 | 27 | 35 | 5 | 0 | 551 |
| B10 | 100-150 | 24 | 35 | 4 | 0 | 256 |
| B10 | 150-200 | 18 | 35 | 2 | 0 | 80 |
| A01 | 0-17 | 15 | 35 | 2 | 0 | 65 |
|  | 17-34 | 14 | 35 | 1 | 0 | 23 |
|  | 34-51 | 28 | 35 | 4 | 0 | 433 |
|  | 50-100 | 25 | 35 | 5 | 0 | 434 |
| A02 | 0-17 | 22 | 35 | 4 | 0 | 197 |
|  | 17-34 | 15 | 35 | 2 | 0 | 15 |
|  | 34-51 | 14 | 35 | 1 | 0 | 17 |
|  | 50-100 | 28 | 35 | 4 | 0 | 291 |
|  | 100-150 | 26 | 35 | 4 | 0 | 203 |
|  | 150-200 | 21 | 35 | 3 | 0 | 119 |
| A03 | 0-17 | 18 | 35 | 2 | 0 | 49 |
|  | 17-34 | 15 | 35 | 2 | 0 | 66 |
|  | 34-51 | 26 | 35 | 5 | 0 | 357 |
|  | 100-150 | 21 | 35 | 4 | 1 | 194 |
|  | 150-200 | 19 | 35 | 3 | 0 | 80 |
| A14 | 0-17 | 17 | 35 | 2 | 0 | 42 |
|  | 17-34 | 14 | 35 | 1 | 0 | 16 |
|  | 34-51 | 14 | 35 | 1 | 0 | 8 |
|  | 50-100 | 27 | 35 | 5 | 0 | 233 |
|  | 100-150 | 24 | 35 | 5 | 0 | 150 |
|  | 150-200 | 20 | 35 | 3 | 0 | 61 |
| A15 | 0-17 | 16 | 35 | 2 | 0 | 60 |
|  | 17-34 | 14 | 35 | 1 | 0 | 58 |
|  | 34-51 | 13 | 35 | 1 | 0 | 136 |
|  | 50-100 | 28 | 35 | 5 | 0 | 269 |
|  | 100-150 | 23 | 35 | 4 | 0 | 65 |
|  | 150-200 | 19 | 35 | 2 | 1 | 345 |
| A16 | 0-17 | 17 | 35 | 1 | 0 | 30 |
|  | 17-34 | 15 | 35 | 2 | 0 | 27 |
|  | 34-51 | 14 | 35 | 1 | 0 | 38 |
|  | 50-100 | 28 | 35 | 4 | 0 | 210 |
|  | 100-150 | 25 | 35 | 4 | 0 | 349 |
| A17 | 0-17 | 20 | 35 | 3 | 0 | 485 |
|  | 17-34 | 17 | 35 | 2 | 0 | 315 |
|  | 34-51 | 15 | 35 | 2 | 0 | 10 |
|  | 50-100 | 28 | 35 | 4 | 0 | 55 |
|  | 100-150 | 28 | 35 | 4 | 0 | 118 |
|  | 150-200 | 25 | 35 | 4 | 0 | 728 |
| A18 | 0-17 | 18 | 35 | 2 | 0 | 227 |
|  | 17-34 | 15 | 35 | 2 | 0 | 72 |
|  | 34-51 | 14 | 35 | 1 | 0 | 248 |
|  | 50-100 | 28 | 35 | 4 | 0 | 571 |
|  | 100-150 | 28 | 35 | 4 | 0 | 460 |
|  | 150-200 | 26 | 35 | 4 | 0 | 294 |
| A19 | 0-17 | 19 | 35 | 2 | 0 | 198 |
|  | 17-34 | 16 | 35 | 2 | 0 | 132 |
|  | 34-51 | 14 | 35 | 1 | 0 | 16 |
|  | 50-100 | 28 | 35 | 4 | 0 | 354 |
|  | 100-150 | 19 | 35 | 2 | 0 | 115 |
|  | 150-200 | 15 | 35 | 2 | 0 | 24 |
| C02 | 0-17 | 13 | 35 | 1 | 0 | 25 |
|  | 17-34 | 27 | 35 | 5 | 0 | 781 |
|  | 50-100 | 25 | 35 | 4 | 0 | 281 |
|  | 100-150 | 23 | 35 | 4 | 1 | 249 |
|  | 150-200 | 19 | 35 | 3 | 0 | 294 |
| C05 | 0-17 | 15 | 35 | 2 | 0 | 32 |
|  | 17-34 | 14 | 35 | 1 | 0 | 81 |
|  | 34-51 | 28 | 35 | 4 | 0 | 1160 |
|  | 50-100 | 27 | 35 | 5 | 0 | 963 |
|  | 100-150 | 23 | 35 | 4 | 1 | 67 |
|  | 150-200 | 15 | 35 | 2 | 0 | 85 |
| C06 | 0-17 | 14 | 35 | 1 | 0 | 15 |
|  | 17-34 | 27 | 35 | 5 | 0 | 592 |
|  | 34-51 | 27 | 35 | 5 | 0 | 294 |
|  | 50-100 | 24 | 35 | 4 | 0 | 169 |
|  | 100-150 | 19 | 35 | 3 | 0 | 161 |
|  | 150-200 | 15 | 35 | 2 | 0 | 86 |
| C08 | 0-17 | 13 | 35 | 1 | 0 | 9 |
|  | 17-34 | 27 | 35 | 5 | 0 | 155 |
|  | 34-51 | 21 | 35 | 4 | 1 | 288 |
|  | 50-100 | 19 | 35 | 2 | 0 | 276 |
|  | 100-150 | 30 | 35 | 3 | 0 | 367 |
| C09 | 0-17 | 27 | 35 | 3 | 2 | 367 |
|  | 17-34 | 23 | 35 | 2 | 2 | 247 |
|  | 34-51 | 19 | 35 | 2 | 1 | 61 |
|  | 50-100 | 29 | 35 | 4 | 0 | 427 |
|  | 100-150 | 27 | 35 | 4 | 1 | 361 |
|  | 150-200 | 24 | 35 | 3 | 3 | 176 |
| C11 | 0-17 | 20 | 35 | 2 | 1 | 28 |
|  | 17-34 | 16 | 35 | 1 | 0 | 151 |
|  | 50-100 | 14 | 35 | 1 | 0 | 82 |
|  | 100-150 | 29 | 35 | 4 | 0 | 299 |
|  | 150-200 | 26 | 35 | 3 | 1 | 305 |
| C12 | 0-17 | 23 | 35 | 2 | 2 | 186 |
|  | 17-34 | 16 | 35 | 1 | 0 | 35 |
|  | 34-51 | 14 | 35 | 1 | 0 | 33 |
|  | 50-100 | 30 | 35 | 4 | 1 | 351 |
|  | 100-150 | 28 | 35 | 4 | 1 | 529 |
|  | 150-200 | 25 | 35 | 3 | 3 | 421 |
| C13 | 0-17 | 20 | 35 | 2 | 1 | 73 |
|  | 17-34 | 15 | 35 | 1 | 0 | 162 |
|  | 34-51 | 14 | 35 | 1 | 0 | 91 |
|  | 50-100 | 31 | 35 | 4 | 1 | 244 |
|  | 100-150 | 29 | 35 | 4 | 1 | 555 |
|  | 150-200 | 26 | 35 | 3 | 2 | 129 |
| C17 | 0-17 | 20 | 35 | 2 | 1 | 69 |
|  | 17-34 | 16 | 35 | 1 | 0 | 66 |
|  | 34-51 | 14 | 35 | 1 | 0 | 106 |
|  | 50-100 | 31 | 35 | 4 | 0 | 419 |
|  | 100-150 | 30 | 35 | 3 | 1 | 187 |
|  | 150-200 | 27 | 35 | 3 | 2 | 171 |
| C18 | 0-17 | 20 | 35 | 2 | 1 | 199 |
|  | 17-34 | 16 | 35 | 1 | 0 | 39 |
|  | 34-51 | 14 | 35 | 1 | 0 | 109 |
|  | 50-100 | 30 | 35 | 4 | 0 | 236 |
|  | 100-150 | 26 | 35 | 3 | 2 | 221 |
|  | 150-200 | 19 | 35 | 2 | 0 | 59 |
| C20 | 0-17 | 15 | 35 | 1 | 0 | 17 |
|  | 17-34 | 14 | 35 | 1 | 0 | 124 |
|  | 34-51 | 31 | 35 | 4 | 0 | 89 |
|  | 50-100 | 26 | 35 | 3 | 2 | 879 |
|  | 100-150 | 21 | 35 | 2 | 2 | 80 |
|  | 150-200 | 16 | 35 | 2 | 0 | 126 |
| C21 | 0-17 | 14 | 35 | 1 | 0 | 64 |
|  | 17-34 | 13 | 35 | 1 | 0 | 64 |
|  | 34-51 | 30 | 35 | 4 | 0 | 1881 |
|  | 50-100 | 26 | 35 | 4 | 1 | 95 |
|  | 100-150 | 20 | 35 | 3 | 2 | 402 |
|  | 150-200 | 15 | 35 | 1 | 0 | 24 |
| 1 | 0-50 | 14 | 35 | 1 | 0 | 45 |
|  | 50-100 | 13 | 35 | 1 | 0 | 83 |
|  | 150-200 | 31 | 36 | 4 | 0 | 72 |
| 2 | 0-50 | 28 | 35 | 4 | 0 | 948 |
|  | 50-100 | 21 | 35 | 4 | 3 | 108 |
|  | 100-150 | 15 | 35 | 2 | 0 | 125 |
|  | 150-200 | 13 | 35 | 1 | 0 | 95 |
| 3 | 0-50 | 31 | 35 | 4 | 0 | 425 |
|  | 50-100 | 27 | 35 | 4 | 1 | 582 |
|  | 100-150 | 21 | 35 | 4 | 3 | 131 |
|  | 150-200 | 16 | 35 | 2 | 1 | 26 |
| 4 | 0-50 | 13 | 35 | 1 | 0 | 314 |
|  | 50-100 | 13 | 35 | 1 | 0 | 49 |
|  | 100-150 | 31 | 36 | 4 | 0 | 295 |
|  | 150-200 | 29 | 35 | 4 | 0 | 176 |
| 5 | 0-50 | 18 | 35 | 2 | 1 | 307 |
|  | 50-100 | 15 | 35 | 1 | 0 | 387 |
|  | 100-150 | 13 | 35 | 1 | 0 | 33 |
|  | 150-200 | 31 | 36 | 4 | 0 | 137 |
| 6 | 0-50 | 30 | 36 | 4 | 0 | 320 |
|  | 50-100 | 25 | 35 | 4 | 1 | 202 |
|  | 100-150 | 19 | 35 | 2 | 1 | 200 |
|  | 150-200 | 20 | 35 | 2 | 0 | 122 |
| 7 | 0-50 | 13 | 35 | 1 | 0 | 115 |
|  | 50-100 | 31 | 35 | 4 | 0 | 116 |
|  | 100-150 | 29 | 35 | 4 | 0 | 376 |
|  | 150-200 | 24 | 35 | 3 | 2 | 156 |
| 8 | 0-50 | 19 | 35 | 1 | 0 | 74 |
|  | 50-100 | 16 | 35 | 1 | 0 | 19 |
|  | 100-150 | 14 | 35 | 1 | 0 | 33 |
|  | 150-200 | 29 | 35 | 4 | 1 | 80 |
| 9 | 0-50 | 25 | 35 | 3 | 3 | 381 |
|  | 50-100 | 20 | 35 | 2 | 0 | 122 |
|  | 100-150 | 30 | 35 | 4 | 0 | 155 |
|  | 150-200 | 29 | 35 | 4 | 1 | 277 |
| 10 | 0-50 | 26 | 35 | 3 | 2 | 233 |
|  | 50-100 | 21 | 35 | 2 | 1 | 144 |
|  | 100-150 | 17 | 35 | 2 | 0 | 157 |
|  | 150-200 | 27 | 35 | 3 | 2 | 79 |
| 11 | 0-50 | 21 | 35 | 2 | 0 | 24 |
|  | 50-100 | 18 | 35 | 2 | 0 | 202 |
|  | 100-150 | 30 | 35 | 4 | 0 | 31 |
|  | 150-200 | 28 | 35 | 3 | 2 | 174 |
| 12 | 0-50 | 24 | 35 | 2 | 1 | 76 |
|  | 50-100 | 20 | 35 | 2 | 0 | 27 |
|  | 100-150 | 17 | 35 | 2 | 0 | 111 |
|  | 150-200 | 16 | 35 | 2 | 0 | 154 |
| 13 | 0-50 | 31 | 35 | 4 | 0 | 343 |
|  | 50-100 | 28 | 35 | 3 | 2 | 274 |
|  | 100-150 | 23 | 35 | 2 | 1 | 157 |
|  | 150-200 | 20 | 35 | 2 | 0 | 353 |
| A1 | A01(CM) | 17 | 35 | 2 | 0 | 64 |
| A4 | A04(CM) | 16 | 35 | 2 | 0 | 129 |
| A7 | A07(CM) | 30 | 35 | 4 | 0 | 364 |
| A10 | A10(CM) | 26 | 35 | 3 | 1 | 787 |
| A13 | A13(CM) | 23 | 35 | 2 | 1 | 15 |
| A16 | A16(CM) | 20 | 35 | 2 | 0 | 14 |
| B1 | B01(CM) | 14 | 35 | 1 | 0 | 149 |
| B4 | B04(CM) | 29 | 35 | 4 | 1 | 185 |
| B7 | B07(CM) | 25 | 35 | 3 | 2 | 464 |
| B10 | B10(CM) | 22 | 35 | 2 | 1 | 1340 |
| B13B | B13(CM) | 19 | 35 | 2 | 0 | 252 |
| E11 | E11(CM) | 15 | 35 | 2 | 0 | 46 |
| E13 | E13(CM) | 14 | 35 | 1 | 0 | 133 |
| E15 | E15(CM) | 23 | 36 | 5 | 0 | 703 |
| E17 | E17(CM) | 17 | 35 | 3 | 1 | 72 |
| E19 | E19(CM) | 23 | 36 | 5 | 1 | 792 |
| E21 | E21(CM) | 19 | 35 | 4 | 1 | 572 |
| E23 | E23(CM) | 23 | 35 | 5 | 2 | 237 |
| E25 | E25(CM) | 17 | 35 | 3 | 1 | 638 |
| E27 | E27(CM) | 14 | 35 | 0 | 0 | 183 |
| E29 | E29(CM) | 23 | 35 | 5 | 0 | 488 |
| E31 | E31(CM) | 19 | 35 | 4 | 1 | 743 |
| E33 | E33(CM) | 14 | 35 | 0 | 0 | 772 |
| E35 | E35(CM) | 22 | 35 | 5 | 3 | 677 |
| E37 | E37(CM) | 18 | 35 | 4 | 1 | 620 |
| C1 | C01(CM) | 13 | 35 | 0 | 0 | 87 |
| C3 | C03(CM) | 20 | 35 | 5 | 1 | 1604 |
| C5 | C05(CM) | 22 | 36 | 5 | 1 | 2001 |
| C7 | C07(CM) | 17 | 35 | 3 | 0 | 499 |
| C9 | C09(CM) | 13 | 35 | 0 | 0 | 304 |
| C11 | C11(CM) | 22 | 35 | 6 | 2 | 351 |
| C13 | C13(CM) | 18 | 35 | 3 | 1 | 1711 |
| C15 | C15(CM) | 14 | 35 | 1 | 0 | 471 |
| C17 | C17(CM) | 23 | 35 | 5 | 0 | 2386 |
| A1 | A01(T) | 18 | 35 | 3 | 1 | 1714 |
| A4 | A04(T) | 14 | 35 | 0 | 0 | 951 |
| A7 | A07(T) | 24 | 36 | 5 | 1 | 2905 |
| A10 | A10(T) | 19 | 35 | 4 | 1 | 601 |
| A13 | A13(T) | 14 | 35 | 1 | 0 | 1142 |
| B1 | B01(T) | 23 | 36 | 5 | 0 | 1474 |
| B4 | B04(T) | 18 | 35 | 4 | 1 | 1768 |
| B7 | B07(T) | 14 | 35 | 1 | 0 | 662 |
| B10 | B10(T) | 23 | 35 | 5 | 0 | 422 |
| B13 | B13(T) | 20 | 35 | 5 | 1 | 927 |
| E11 | E11(T) | 17 | 35 | 3 | 0 | 544 |
| E13 | E13(T) | 23 | 35 | 5 | 0 | 1370 |
| E15 | E15(T) | 20 | 35 | 5 | 1 | 61 |
| E17 | E17(T) | 14 | 35 | 0 | 0 | 422 |
| E19 | E10(T) | 23 | 35 | 5 | 0 | 2029 |
| E21 | E21(T) | 20 | 35 | 5 | 0 | 983 |
| E23 | E23(T) | 14 | 35 | 0 | 0 | 369 |
| E25 | E25(T) | 24 | 35 | 5 | 0 | 422 |
| E27 | E27(T) | 19 | 35 | 4 | 1 | 776 |
| E29 | E29(T) | 14 | 35 | 0 | 0 | 109 |
| E31 | E31(T) | 24 | 35 | 5 | 1 | 631 |
| E33 | E33(T) | 18 | 35 | 2 | 1 | 425 |
| E35 | E35(T) | 14 | 35 | 0 | 0 | 1904 |
| E37 | E37(T) | 24 | 35 | 5 | 0 | 561 |
| C1 | C01(T) | 21 | 35 | 5 | 1 | 946 |
| C3 | C03(T) | 19 | 35 | 4 | 1 | 416 |
| C5 | C05(T) | 24 | 35 | 5 | 0 | 1392 |
| C7 | C07(T) | 19 | 35 | 4 | 1 | 949 |
| C9 | C09(T) | 14 | 35 | 1 | 0 | 784 |
| C11 | C11(T) | 24 | 35 | 5 | 0 | 1753 |
| C13 | C13(T) | 20 | 35 | 3 | 1 | 917 |
| C15 | C15(T) | 15 | 35 | 0 | 0 | 404 |
| C17 | C17(T) | 25 | 35 | 5 | 0 | 784 |
| A1 | A01(ZMO) | 20 | 35 | 3 | 1 | 697 |
| A7 | A07(ZMO) | 15 | 35 | 0 | 0 | 297 |
| A10 | A10(ZMO) | 25 | 35 | 5 | 1 | 1394 |
| A13 | A13(ZMO) | 21 | 35 | 2 | 3 | 977 |
| B1 | B01(ZMO) | 16 | 35 | 0 | 0 | 152 |
| B4 | B04(ZMO) | 26 | 35 | 5 | 1 | 858 |
| B7 | B07(ZMO) | 23 | 35 | 4 | 2 | 639 |
| B10 | B10(ZMO) | 17 | 35 | 0 | 0 | 1211 |
| B13 | B13(ZMO) | 26 | 35 | 5 | 0 | 1024 |
| E11 | E11(ZMO) | 21 | 35 | 3 | 2 | 404 |
| E13 | E13(ZMO) | 15 | 35 | 0 | 1 | 1194 |
| E15 | E15(ZMO) | 25 | 35 | 5 | 1 | 1822 |
| E17 | E17(ZMO) | 21 | 35 | 3 | 2 | 798 |
| E19 | E19(ZMO) | 26 | 35 | 5 | 1 | 559 |
| E21 | E21(ZMO) | 23 | 35 | 4 | 2 | 327 |
| E23 | E23(ZMO) | 24 | 35 | 6 | 6 | 952 |
| E25 | E25(ZMO) | 20 | 35 | 3 | 3 | 764 |
| E27 | E27(ZMO) | 17 | 35 | 1 | 1 | 1360 |
| E29 | E29(ZMO) | 24 | 35 | 5 | 0 | 827 |
| E31 | E31(ZMO) | 19 | 35 | 3 | 1 | 246 |
| E33 | E33(ZMO) | 14 | 35 | 0 | 0 | 815 |
| E35 | E35(ZMO) | 23 | 35 | 5 | 2 | 529 |
| E37 | E37(ZMO) | 17 | 35 | 2 | 0 | 60 |
| C1 | C01(ZMO) | 14 | 35 | 0 | 0 | 267 |
| C3 | C03(ZMO) | 24 | 35 | 5 | 0 | 923 |
| C5 | C05(ZMO) | 23 | 35 | 5 | 1 | 264 |
| C9 | C09(ZMO) | 14 | 35 | 0 | 0 | 1063 |
| C11 | C11(ZMO) | 24 | 35 | 5 | 0 | 301 |
| C13 | C13(ZMO) | 21 | 35 | 5 | 1 | 124 |
| C15 | C15(ZMO) | 14 | 35 | 1 | 0 | 638 |
| C17 | C17(ZMO) | 24 | 35 | 5 | 0 | 750 |
