# Supplementary material for: Effects of Geostrophic Kinetic Energy on the Distribution of Mesopelagic Fish Larvae in the Southern Gulf of California in Summer/Fall Stratified Seasons
Source: PLoS One. 2016 Oct 19;11(10):e0164900. doi: 10.1371/journal.pone.0164900 (PMC5070878; doi:10.1371/journal.pone.0164900)
Supplement: S2 Table — A Number of fish larvae in lines with high geostrophic kinetic energy flux. B Table Average of environmental variables in lines with high geostrophic kinetic energy flux. (DOCX) [file pone.0164900.s002.docx]

S2A Table Number of fish larvae in lines with high energy anomalies

| Station | Strata | *Benthosema panamense* | *Diogenichthys laternatus* | *Triphoturus mexicanus* | *Vinciguerria lucetia* |
| --- | --- | --- | --- | --- | --- |
| 4 | 0-17 | 0 | 0 | 0 | 0 |
|  | 17-34 | 0 | 0 | 0 | 1 |
|  | 34-51 | 5 | 0 | 21 | 4 |
|  | 50-100 | 1 | 0 | 1 | 1 |
|  | 100-150 | 0 | 1 | 0 | 0 |
|  | 150-200 | 0 | 1 | 2 | 0 |
| 5 | 0-17 | 1 | 0 | 0 | 0 |
|  | 17-34 | 1 | 0 | 0 | 0 |
|  | 34-51 | 13 | 0 | 26 | 14 |
|  | 50-100 | 1 | 0 | 1 | 0 |
|  | 100-150 | 3 | 0 | 6 | 0 |
|  | 150-200 | 0 | 0 | 1 | 0 |
| 6 | 0-17 | 2 | 0 | 1 | 7 |
|  | 17-34 | 22 | 0 | 7 | 73 |
|  | 34-51 | 3 | 0 | 23 | 9 |
|  | 50-100 | 0 | 3 | 14 | 13 |
|  | 100-150 | 3 | 1 | 3 | 5 |
|  | 150-200 | 1 | 0 | 13 | 10 |
| 7 | 0-17 | 4 | 0 | 5 | 41 |
|  | 17-34 | 5 | 0 | 7 | 62 |
|  | 34-51 | 11 | 0 | 21 | 9 |
|  | 50-100 | 0 | 0 | 6 | 3 |
|  | 100-150 | 4 | 0 | 6 | 5 |
|  | 150-200 | 0 | 0 | 2 | 0 |
| 8 | 0-17 | 0 | 0 | 0 | 16 |
|  | 17-34 | 17 | 0 | 16 | 169 |
|  | 34-51 | 7 | 5 | 5 | 96 |
|  | 50-100 | 0 | 3 | 4 | 6 |
|  | 100-150 | 0 | 37 | 1 | 18 |
|  | 150-200 | 0 | 3 | 0 | 10 |
| 9 | 0-17 | 0 | 0 | 0 | 2 |
|  | 17-34 | 1 | 0 | 4 | 15 |
|  | 34-51 | 7 | 6 | 24 | 133 |
|  | 50-100 | 0 | 4 | 5 | 7 |
|  | 100-150 | 0 | 0 | 0 | 4 |
|  | 150-200 | 1 | 2 | 1 | 10 |
| 10 | 0-17 | 0 | 0 | 0 | 0 |
|  | 17-34 | 18 | 0 | 8 | 10 |
|  | 50-100 | 3 | 21 | 1 | 36 |
| 11 | 0-17 | 0 | 0 | 0 | 5 |
|  | 17-34 | 18 | 0 | 4 | 139 |
|  | 34-51 | 0 | 9 | 1 | 8 |
|  | 50-100 | 0 | 3 | 2 | 7 |
|  | 100-150 | 0 | 3 | 3 | 8 |
| 12 | 0-17 | 0 | 0 | 0 | 9 |
|  | 17-34 | 6 | 0 | 7 | 31 |
|  | 34-51 | 0 | 1 | 0 | 19 |
|  | 50-100 | 1 | 6 | 10 | 96 |
|  | 100-150 | 0 | 78 | 3 | 26 |
|  | 150-200 | 0 | 9 | 1 | 46 |
| 13 | 0-17 | 1 | 0 | 0 | 41 |
|  | 34-51 | 7 | 18 | 17 | 410 |
|  | 50-100 | 0 | 5 | 4 | 28 |
|  | 100-150 | 0 | 5 | 0 | 2 |
| 1 | 15-30 | 0 | 1 | 0 | 4 |
|  | 30-45 | 71 | 13 | 7 | 55 |
|  | 50-100 | 0 | 0 | 0 | 3 |
|  | 100-150 | 1 | 5 | 2 | 2 |
|  | 150-200 | 1 | 4 | 0 | 3 |
| 2 | 0-15 | 0 | 0 | 1 | 2 |
|  | 15-30 | 0 | 1 | 0 | 5 |
|  | 30-45 | 0 | 0 | 0 | 3 |
|  | 50-100 | 0 | 69 | 0 | 2 |
| 3 | 0-15 | 1 | 0 | 0 | 31 |
|  | 50-100 | 0 | 0 | 0 | 25 |
|  | 100-150 | 0 | 4 | 0 | 1 |
|  | 150-200 | 1 | 6 | 0 | 4 |
| 4 | 0-15 | 1 | 0 | 0 | 57 |
|  | 15-30 | 0 | 7 | 3 | 152 |
|  | 30-45 | 2 | 2 | 1 | 118 |
|  | 50-100 | 0 | 6 | 1 | 14 |
|  | 100-150 | 0 | 28 | 1 | 0 |
|  | 150-200 | 0 | 2 | 0 | 1 |
| 1 | 0-50 | 19 | 0 | 2 | 6 |
|  | 50-100 | 11 | 2 | 3 | 3 |
|  | 150-200 | 1 | 3 | 2 | 0 |
| 2 | 0-50 | 5 | 0 | 2 | 0 |
|  | 50-100 | 13 | 0 | 1 | 3 |
|  | 100-150 | 2 | 1 | 0 | 0 |
|  | 150-200 | 1 | 5 | 0 | 0 |
| 3 | 0-50 | 3 | 2 | 1 | 4 |
|  | 50-100 | 5 | 8 | 1 | 2 |
|  | 100-150 | 2 | 0 | 0 | 0 |
|  | 150-200 | 0 | 4 | 0 | 0 |
| 4 | 0-50 | 2 | 0 | 2 | 12 |
|  | 50-100 | 0 | 0 | 1 | 6 |
|  | 100-150 | 3 | 0 | 3 | 12 |
|  | 150-200 | 3 | 0 | 0 | 5 |
| 5 | 0-50 | 5 | 0 | 4 | 12 |
|  | 50-100 | 4 | 2 | 2 | 4 |
|  | 100-150 | 7 | 10 | 10 | 16 |
|  | 150-200 | 2 | 0 | 0 | 1 |
| 6 | 0-50 | 5 | 0 | 2 | 15 |
|  | 50-100 | 11 | 0 | 3 | 9 |
|  | 100-150 | 0 | 0 | 0 | 1 |
|  | 150-200 | 1 | 4 | 1 | 3 |
| 7 | 0-50 | 2 | 0 | 0 | 4 |
|  | 50-100 | 3 | 0 | 5 | 6 |
|  | 100-150 | 1 | 1 | 6 | 9 |
|  | 150-200 | 8 | 2 | 11 | 6 |
| 8 | 0-50 | 0 | 0 | 1 | 3 |
|  | 50-100 | 0 | 1 | 2 | 0 |
|  | 100-150 | 1 | 1 | 0 | 0 |
|  | 150-200 | 0 | 1 | 0 | 0 |
| 9 | 0-50 | 14 | 3 | 1 | 0 |
|  | 50-100 | 12 | 2 | 0 | 0 |
|  | 100-150 | 2 | 1 | 0 | 0 |
|  | 150-200 | 0 | 7 | 1 | 0 |
| 10 | 0-50 | 9 | 0 | 5 | 7 |
|  | 50-100 | 6 | 1 | 3 | 2 |
|  | 100-150 | 6 | 1 | 7 | 12 |
|  | 150-200 | 0 | 0 | 0 | 1 |
| 11 | 0-50 | 32 | 0 | 26 | 7 |
|  | 50-100 | 10 | 1 | 19 | 1 |
|  | 100-150 | 0 | 0 | 2 | 0 |
|  | 150-200 | 7 | 2 | 9 | 3 |
| 12 | 0-50 | 6 | 0 | 12 | 15 |
|  | 50-100 | 0 | 1 | 9 | 0 |
|  | 100-150 | 10 | 0 | 8 | 29 |
|  | 150-200 | 0 | 0 | 3 | 1 |
| 13 | 0-50 | 16 | 0 | 3 | 21 |
|  | 50-100 | 1 | 10 | 3 | 7 |
|  | 100-150 | 1 | 0 | 0 | 2 |
|  | 150-200 | 7 | 2 | 1 | 1 |

S2B Table Average of environmental variables in lines with high energy anomalies

| Station | Strata | Temperature | Salinity | Dissolved oxygen | Fluorescence | Zooplankton displacement biomass |
| --- | --- | --- | --- | --- | --- | --- |
| 4 | 0-17 | 25 | 35 | 5 | 0 | 89 |
|  | 17-34 | 21 | 35 | 4 | 0 | 0 |
|  | 34-51 | 16 | 35 | 2 | 0 | 46 |
|  | 50-100 | 14 | 35 | 1 | 0 | 31 |
|  | 100-150 | 13 | 35 | 1 | 0 | 49 |
|  | 150-200 | 28 | 35 | 4 | 0 | 70 |
| 5 | 0-17 | 26 | 35 | 5 | 0 | 28 |
|  | 17-34 | 21 | 35 | 3 | 0 | 0 |
|  | 34-51 | 17 | 35 | 2 | 0 | 112 |
|  | 50-100 | 28 | 35 | 5 | 0 | 873 |
|  | 100-150 | 17 | 35 | 2 | 0 | 13 |
|  | 150-200 | 13 | 35 | 1 | 0 | 74 |
| 6 | 0-17 | 11 | 35 | 0 | 0 | 14 |
|  | 17-34 | 28 | 35 | 4 | 0 | 398 |
|  | 34-51 | 28 | 35 | 5 | 0 | 437 |
|  | 50-100 | 24 | 35 | 4 | 0 | 165 |
|  | 100-150 | 18 | 35 | 2 | 0 | 96 |
|  | 150-200 | 13 | 35 | 1 | 0 | 165 |
| 7 | 0-17 | 12 | 35 | 0 | 0 | 43 |
|  | 17-34 | 27 | 35 | 4 | 0 | 153 |
|  | 34-51 | 19 | 35 | 2 | 0 | 76 |
|  | 50-100 | 13 | 35 | 1 | 0 | 39 |
|  | 100-150 | 28 | 35 | 4 | 0 | 178 |
|  | 150-200 | 20 | 35 | 2 | 0 | 122 |
| 8 | 0-17 | 15 | 35 | 1 | 0 | 49 |
|  | 17-34 | 13 | 35 | 1 | 0 | 70 |
|  | 34-51 | 29 | 35 | 4 | 0 | 130 |
|  | 50-100 | 21 | 35 | 2 | 0 | 46 |
|  | 100-150 | 15 | 35 | 1 | 0 | 37 |
|  | 150-200 | 13 | 35 | 0 | 0 | 34 |
| 9 | 0-17 | 29 | 35 | 4 | 0 | 158 |
|  | 17-34 | 21 | 35 | 2 | 0 | 3 |
|  | 34-51 | 14 | 35 | 1 | 0 | 47 |
|  | 50-100 | 13 | 35 | 0 | 0 | 29 |
|  | 100-150 | 29 | 35 | 4 | 1 | 176 |
|  | 150-200 | 22 | 35 | 2 | 0 | 65 |
| 10 | 0-17 | 15 | 35 | 1 | 0 | 139 |
|  | 17-34 | 13 | 35 | 0 | 0 | 47 |
|  | 50-100 | 29 | 35 | 4 | 1 | 270 |
| 11 | 0-17 | 21 | 35 | 2 | 0 | 85 |
|  | 17-34 | 14 | 35 | 0 | 0 | 30 |
|  | 34-51 | 12 | 35 | 0 | 0 | 52 |
|  | 50-100 | 28 | 35 | 4 | 1 | 196 |
|  | 100-150 | 21 | 35 | 2 | 0 | 71 |
| 12 | 0-17 | 15 | 35 | 1 | 0 | 68 |
|  | 17-34 | 13 | 35 | 0 | 0 | 44 |
|  | 34-51 | 28 | 35 | 4 | 0 | 130 |
|  | 50-100 | 21 | 35 | 2 | 0 | 118 |
|  | 100-150 | 15 | 35 | 1 | 0 | 61 |
|  | 150-200 | 13 | 35 | 0 | 0 | 121 |
| 13 | 0-17 | 28 | 35 | 4 | 1 | 140 |
|  | 34-51 | 20 | 35 | 2 | 0 | 82 |
|  | 50-100 | 14 | 35 | 1 | 0 | 125 |
|  | 100-150 | 12 | 35 | 0 | 0 | 45 |
| A16 | 0-17 | 27 | 35 | 4 | 1 | 284 |
|  | 17-34 | 18 | 35 | 2 | 0 | 83 |
|  | 34-51 | 14 | 35 | 1 | 0 | 85 |
| 1 | 15-30 | 13 | 35 | 0 | 0 | 39 |
|  | 30-45 | 30 | 35 | 4 | 0 | 364 |
|  | 50-100 | 29 | 35 | 3 | 1 | 174 |
|  | 100-150 | 23 | 35 | 2 | 2 | 136 |
|  | 150-200 | 30 | 35 | 4 | 0 | 251 |
| 2 | 0-15 | 29 | 35 | 4 | 0 | 303 |
|  | 15-30 | 24 | 35 | 3 | 2 | 208 |
|  | 30-45 | 18 | 35 | 2 | 0 | 94 |
|  | 50-100 | 16 | 35 | 1 | 0 | 182 |
| 3 | 0-15 | 14 | 35 | 1 | 0 | 82 |
|  | 50-100 | 30 | 35 | 4 | 0 | 242 |
|  | 100-150 | 27 | 35 | 3 | 1 | 345 |
|  | 150-200 | 22 | 35 | 2 | 1 | 57 |
| 4 | 0-15 | 18 | 35 | 2 | 0 | 46 |
|  | 15-30 | 15 | 35 | 1 | 0 | 217 |
|  | 30-45 | 27 | 35 | 3 | 1 | 444 |
|  | 50-100 | 20 | 35 | 2 | 2 | 233 |
|  | 100-150 | 17 | 35 | 2 | 1 | 133 |
|  | 150-200 | 14 | 35 | 1 | 0 | 393 |
| 1 | 0-50 | 13 | 35 | 1 | 0 | 12 |
|  | 50-100 | 28 | 35 | 4 | 0 | 492 |
|  | 150-200 | 22 | 35 | 4 | 2 | 405 |
| 2 | 0-50 | 16 | 35 | 2 | 0 | 390 |
|  | 50-100 | 14 | 35 | 1 | 0 | 474 |
|  | 100-150 | 13 | 35 | 1 | 0 | 107 |
|  | 150-200 | 28 | 35 | 4 | 0 | 574 |
| 3 | 0-50 | 15 | 35 | 2 | 0 | 243 |
|  | 50-100 | 31 | 36 | 4 | 0 | 621 |
|  | 100-150 | 28 | 35 | 4 | 0 | 810 |
|  | 150-200 | 21 | 35 | 3 | 3 | 3819 |
| 4 | 0-50 | 15 | 35 | 2 | 0 | 79 |
|  | 50-100 | 13 | 35 | 1 | 0 | 168 |
|  | 100-150 | 28 | 35 | 4 | 0 | 292 |
|  | 150-200 | 21 | 35 | 4 | 3 | 103 |
| 5 | 0-50 | 16 | 35 | 2 | 0 | 279 |
|  | 50-100 | 13 | 35 | 1 | 0 | 1 |
|  | 100-150 | 12 | 35 | 1 | 0 | 194 |
|  | 150-200 | 30 | 35 | 4 | 0 | 2165 |
| 6 | 0-50 | 22 | 35 | 4 | 3 | 150 |
|  | 50-100 | 16 | 35 | 2 | 0 | 114 |
|  | 100-150 | 13 | 35 | 1 | 0 | 294 |
|  | 150-200 | 30 | 35 | 4 | 0 | 714 |
| 7 | 0-50 | 24 | 35 | 4 | 4 | 204 |
|  | 50-100 | 21 | 35 | 3 | 4 | 210 |
|  | 100-150 | 16 | 35 | 2 | 0 | 60 |
|  | 150-200 | 14 | 35 | 1 | 0 | 24 |
| 8 | 0-50 | 12 | 35 | 1 | 0 | 179 |
|  | 50-100 | 31 | 36 | 4 | 0 | 66 |
|  | 100-150 | 18 | 35 | 2 | 1 | 57 |
|  | 150-200 | 30 | 35 | 4 | 0 | 368 |
| 9 | 0-50 | 28 | 35 | 4 | 0 | 450 |
|  | 50-100 | 24 | 35 | 3 | 2 | 231 |
|  | 100-150 | 19 | 35 | 2 | 0 | 36 |
|  | 150-200 | 15 | 35 | 1 | 0 | 162 |
| 10 | 0-50 | 30 | 35 | 4 | 1 | 351 |
|  | 50-100 | 28 | 35 | 4 | 1 | 529 |
|  | 100-150 | 25 | 35 | 3 | 3 | 421 |
|  | 150-200 | 19 | 35 | 2 | 1 | 73 |
| 11 | 0-50 | 15 | 35 | 1 | 0 | 66 |
|  | 50-100 | 14 | 35 | 1 | 0 | 91 |
|  | 100-150 | 29 | 35 | 4 | 1 | 555 |
|  | 150-200 | 26 | 35 | 3 | 2 | 129 |
| 12 | 0-50 | 20 | 35 | 2 | 1 | 69 |
|  | 50-100 | 16 | 35 | 1 | 0 | 39 |
|  | 100-150 | 14 | 35 | 1 | 0 | 106 |
|  | 150-200 | 30 | 35 | 3 | 1 | 139 |
| 13 | 0-50 | 27 | 35 | 3 | 2 | 171 |
|  | 50-100 | 20 | 35 | 2 | 1 | 199 |
|  | 100-150 | 16 | 35 | 1 | 0 | 31 |
|  | 150-200 | 14 | 35 | 1 | 0 | 121 |
